# From acidophilic to ornithogenic: microbial community dynamics in moss banks altered by gentoo penguins

**DOI:** 10.3389/fmicb.2024.1362975

**Published:** 2024-03-07

**Authors:** Yevheniia Prekrasna-Kviatkovska, Ivan Parnikoza, Anna Yerkhova, Olesia Stelmakh, Mariia Pavlovska, Marta Dzyndra, Oleksandr Yarovyi, Evgen Dykyi

**Affiliations:** ^1^Biology and Ecology Department, State Institution National Antarctic Scientific Center, Kyiv, Ukraine; ^2^Department of Cell Population Genetics, Institute of Molecular Biology and Genetics, Kyiv, Ukraine; ^3^Faculty of Natural Science, National University of “Kyiv-Mohyla Academy”, Kyiv, Ukraine; ^4^Biomedical Institute, Open International University of Human Development Ukraine, Kyiv, Ukraine; ^5^Faculty of Molecular Biology and Biotechnology, Kyiv Academic University, Kyiv, Ukraine; ^6^Faculty of Plant Protection, Biotechnology and Ecology, National University of Life and Environmental Sciences of Ukraine, Kyiv, Ukraine

**Keywords:** peat microbial communities, climate change, ornithogenic impact, Antarctica, moss banks

## Abstract

**Introduction:**

The study explores the indirect impact of climate change driven by gentoo’s penguin colonization pressure on the microbial communities of moss banks formed by Tall moss turf subformation in central maritime Antarctica.

**Methods:**

Microbial communities and chemical composition of the differently affected moss banks (Unaffected, Impacted and Desolated) located on Galindez Island and Сape Tuxen on the mainland of Kyiv Peninsula were analyzed.

**Results:**

The native microbiota of the moss banks’ peat was analyzed for the first time, revealing a predominant presence of Acidobacteria (32.2 ± 14.4%), followed by Actinobacteria (15.1 ± 4.0%) and Alphaproteobacteria (9.7 ± 4.1%). Penguin colonization and subsequent desolation of moss banks resulted in an increase in peat pH (from 4.7 ± 0.05 to 7.2 ± 0.6) and elevated concentrations of soluble nitrogen (from 1.8 ± 0.4 to 46.9 ± 2.1 DIN, mg/kg) and soluble phosphorus compounds (from 3.6 ± 2.6 to 20.0 ± 1.8 DIP, mg/kg). The contrasting composition of peat and penguin feces led to the elimination of the initial peat microbiota, with an increase in Betaproteobacteria (from 1.3 ± 0.8% to 30.5 ± 23%) and Bacteroidota (from 5.5 ± 3.7% to 19.0 ± 3.7%) proportional to the intensity of penguins’ impact, accompanied by a decrease in community diversity. Microbial taxa associated with birds’ guts, such as *Gottschalkia* and *Tissierella*, emerged in Impacted and Desolated moss banks, along with bacteria likely benefiting from eutrophication. The changes in the functional capacity of the penguin-affected peat microbial communities were also detected. The nitrogen-cycling genes that regulate the conversion of urea into ammonia, nitrite oxide, and nitrate oxide (*ureC*, *amoA*, *nirS*, *nosZ*, *nxrB*) had elevated copy numbers in the affected peat. Desolated peat samples exhibit the highest nitrogen-cycle gene numbers, significantly differing from Unaffected peat (*p* < 0.05).

**Discussion:**

The expansion of gentoo penguins induced by climate change led to the replacement of acidophilic microbiomes associated with moss banks, shaping a new microbial community influenced by penguin guano’s chemical and microbial composition.

## 1 Introduction

The Western Antarctic Peninsula has experienced the fastest climate change than anywhere on Earth during the last 50 years. Such rapid changes inevitably affect the conditions for the functioning of Antarctica’s terrestrial and marine biota (Smith et al., 1999; Vaughan et al., 2003; Bromwich et al., 2013). While climate changes threaten some species in the region, such as Adélie penguin or krill (Atkinson et al., 2004; Emmerson and Southwell, 2022), others benefit from these alterations. In the latter context, species adapted to the comparatively moderate subantarctic conditions can be mentioned. Examples include the growth of *Pygoscelis papua* or gentoo penguin population (Lynch et al., 2012; Korczak-Abshire et al., 2021), the salp expansion in the Southern Ocean (Atkinson et al., 2004), and the spread of crabs from Patagonia to the Southern Ocean (Hellberg et al., 2019).

For species such as gentoo penguins, salps, or krill, the impact of climate change—whether positive or negative—is directly linked to rising temperatures, ice retreat, or the expanding areas available for colonization. Nevertheless, climatic changes extend beyond direct impacts and have large-scale indirect consequences on Antarctic biota.

The indirect impact of climate change is reliably illustrated by the influence of the Southward expansion of the gentoo penguin (Lynch et al., 2012; LaRue et al., 2013; Younger et al., 2015). An example of such expansion is the first appearance of gentoo penguins on Galindez Island in central maritime Antarctica in 2006, followed by rapid colonization and population growth (Parnikoza et al., 2018). Though penguins can often occupy barren areas released after recent glacier retreat (LaRue et al., 2013; Younger et al., 2015), gentoo penguins also establish colonies on moss banks, which are the most developed forms of the Tall moss turf subformation, as it happened on Galindez Island. These cryptogamic communities are exclusive to the maritime Antarctic and are composed of one or two moss species capable of accumulating peat: *Polytrichum strictum* Brid. and *Chorisodontium aciphyllum* (Hook.f. & Wilson) Broth. (Wierzgoń et al., 2023). Eutrophication resulting from gentoo penguins’ colonization leads to the desolation of moss banks and the flourishing of the nitrophilic *Prasiola crispa* (Lightfoot) Kützing (Parnikoza et al., 2018; Wierzgoń et al., 2023). It induces changes in the structure of plant communities, followed by a loss of diversity and the elimination of species poorly adapted to high eutrophication levels. This scenario was observed on Galindez Island, devoid of gentoo colonies until the 2007/08 season (Parnikoza et al., 2018). The authors have observed a similar process on Cape Tuxen since 2014. The ongoing loss of diversity on Galindez Island and other localities caused by ornithogenic eutrophication continues to unfold.

Besides the ornithogenic effect observable to the naked eye, penguin colonization is likely to affect the chemical and microbiological composition of the peat accumulated beneath the plant community. The peat in moss banks in Antarctica possesses distinctive characteristics, setting it apart from other substrates in the region, including acidity, aerobicity, slow decomposition, and the accumulation of partially decomposed plant organic matter (Fenton, 1980; Parnikoza et al., 2017). These unique properties likely support specific microbial communities. As illustrated earlier, penguin feces alter the composition of the soil microbial community (Wang et al., 2015; Santamans et al., 2017). The guano of penguins is highly enriched in nitrogen compounds, primarily represented by urea, proteins, and ammonium (Lindeboom, 1984; Zhu et al., 2009; Crittenden et al., 2015; Lorrain et al., 2017). Studies of the mineral and ornithogenic soils in Cape Hallet and Cape Bird in the Ross Sea region revealed the enrichment in organic carbon, nitrogen, phosphorus and microbial biomass (Aislabie et al., 2009). Similarly, there were enriched concentrations of total carbon, total organic carbon and total nitrogen content in ornithogenic soils formed by *P. papua* and *P. antarctica* compared to the mineral soils on the southern coast of the Barton Peninsula of King George Island (Kim et al., 2012), and water content, organic matter content, and phosphorus content were higher in bird-impacted soil from Fildes Peninsula, Ardley Peninsula, and Livingston Island (Ramírez-Fernández et al., 2021). Alongside nutrients, the microbiota from penguins’ intestines is released into the Antarctic environment (Grzesiak et al., 2020). Bacterial community composition exhibited significant distinctions between soils influenced by penguins’ activity and mineral soils on the southern coast of the Barton Peninsula (Kim et al., 2012). According to Aislabie et al. (2009) *Firmicutes* and *Psychrobacter* dominated the nest sites of the Adelie penguins, while *Xantomonadaceae*, *Rhodanobacter*, *Dokdonella*, and *Lysobacter* dominated the abandoned sites. Analysis of the bacterial metagenomically assembled genomes (MAGs) (Ramírez-Fernández et al., 2021) revealed that bird impact on Antarctic soil changes the functional activity of microbes. In particular, *nirK* and *nosZ* genes (encoding for nitrite reductase and nitrous oxide reductase) were more significantly abundant in bird-impacted soils than soils without animal influence. Ornithogenic eutrophication and the input of gut microbiota are likely to alter moss banks’ peat and the specific microbial communities that inhabit this substrate.

In Antarctica, microbial communities typically constitute the dominant biomass component of terrestrial ecosystems. They play a pivotal role in controlling the biological flux of carbon, nutrients, and energy (Wynn-Williams, 1996), thereby influencing the functioning and development of terrestrial ecosystems (Yergeau and Kowalchuk, 2008; Teixeira et al., 2010; Yergeau et al., 2012). Given the crucial role of microorganisms in Antarctic terrestrial life, penguin-induced eutrophication can lead to the replacement of the peat microbiota, impairment or changes in its functional capabilities. The intense input of penguin guano rich in nitrogen compounds is likely to alter the functioning of nitrogen-cycling bacteria, initially leading to enrichment in nitrogen-cycling taxa.

Herewith, the study’s objective was to investigate the gentoo penguin’s increasing colonization influence on the microbial communities of peat moss banks in central maritime Antarctica. The particular tasks of the study were: (i) estimate changes in the chemical parameters of the peat; (ii) evaluate the changes in microbial composition as the result of the penguins’ colonization; (iii) compare the abundance of the microbial nitrogen-cycling genes in the peat differentially affected by penguins.

## 2 Materials and methods

### 2.1 Study sites

**Figure 1:**
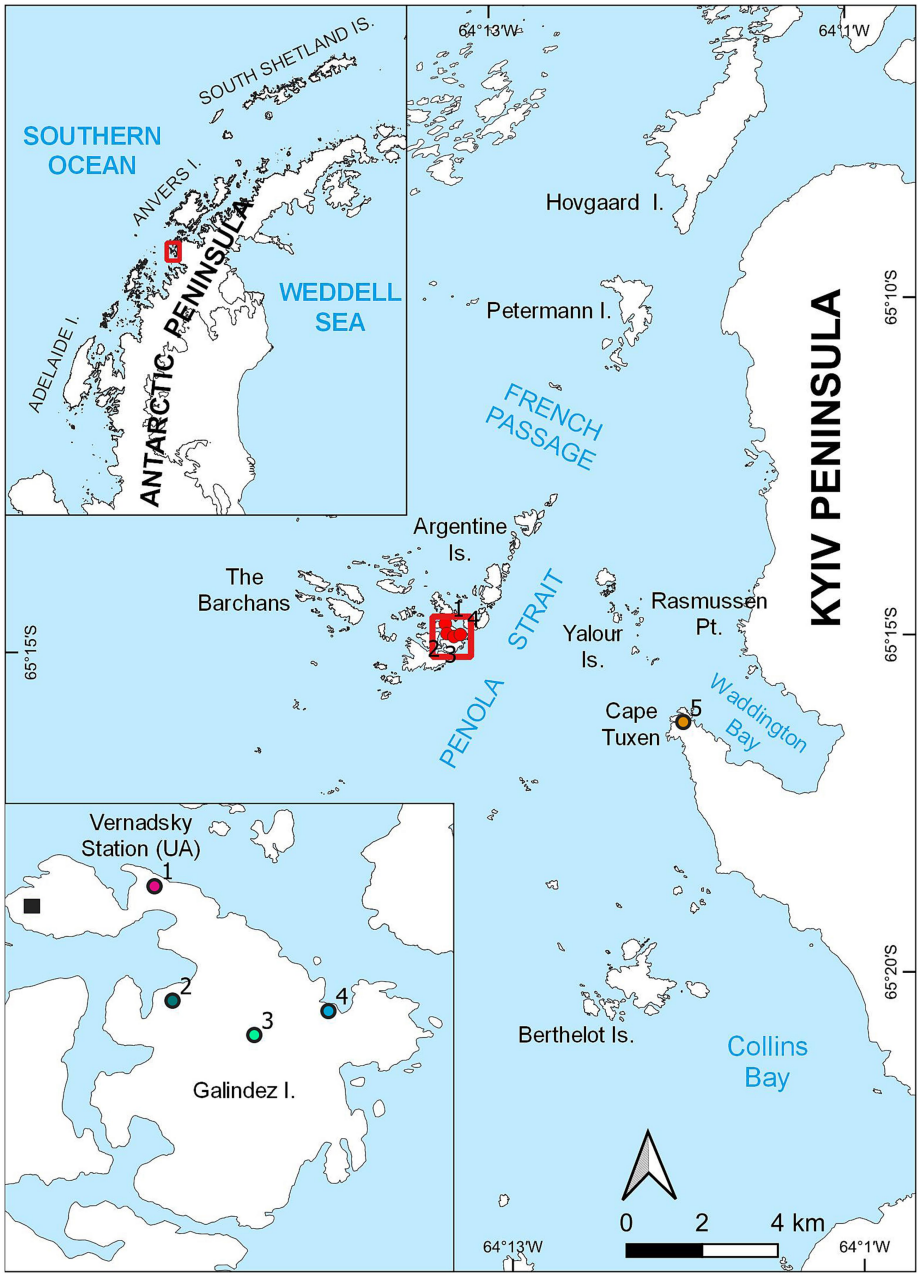
Locations of the moss banks located on Galindez Island and Сape Tuxen on the mainland of Kyiv Peninsula, where samples were collected.

Several moss banks influenced by penguin colonies expansion have been studied. These moss banks, namely moss banks 1 and 4 are located on Galindez Island, Argentine Islands (Figure 1, points 1 and 4 respectively). The other moss bank 5 is located on the Сape Tuxen on the mainland of Kyiv Peninsula (Figure 1, point 5). All studied moss banks were formed mainly by *Polytrichum strictum* (Schrader) but had significant diversity (Wierzgoń et al., 2023).

Penguin colonies appeared on the moss bank 1 in 2019, though birds abandoned this territory afterwards, leaving single nests. Both moss banks 4 and 5 were colonized by *P. papua* in 2020 and were considered newly penguin-disturbed territories at the moment of sampling. Moss banks 2 and 3 located on Galindez Island were not disturbed by penguins at the moment of sampling.

**Figure 2:**
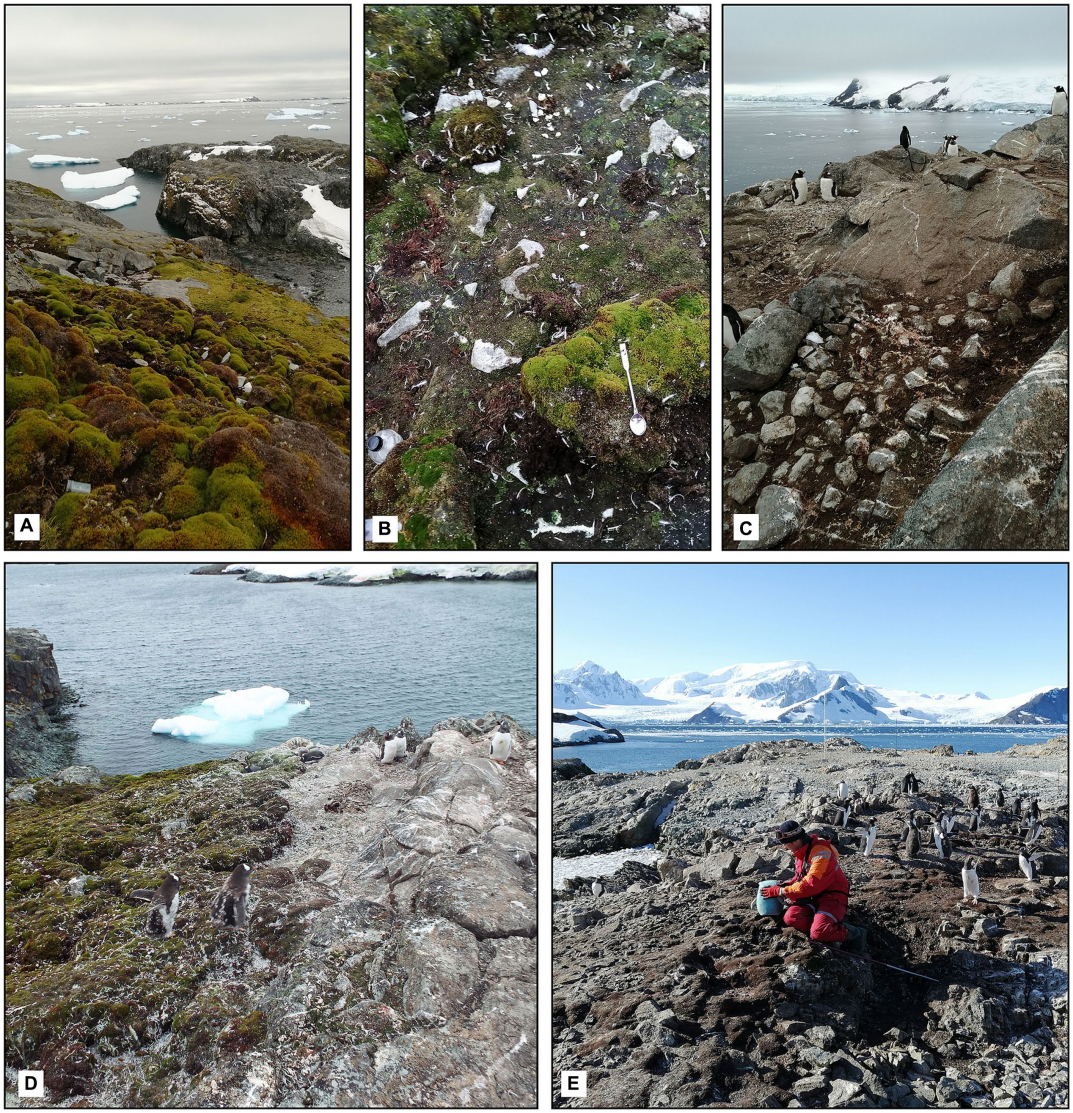
Levels of the ornithogenic impact on the moss banks: **(A)** Unaffected moss bank, moss bank 5; **(B)** impacted moss bank, moss bank 5; **(C)** desolated moss bank covered by guano, moss bank 5; **(D)** expansion of the penguin colony on the moss bank 4; **(E)** desolated part of moss bank 4 (2020).

Moss banks were not evenly affected by penguins’ direct impact, such as colonization, trampling, and excretion. The moss banks that were not affected directly by the birds had no visual sign of the impact (Figure 2). We define 3 degrees of moss bank colonization by penguins (Figure 2).

- Unaffected: Intact moss banks that show no direct and visible penguin influence. Samples of unaffected peat were collected from moss banks 2 and 3 on Galindez Island [Figure 1, points 2 and 3, respectively (Wierzgoń et al., 2023)] in addition to moss banks 1, 4, and 5. These moss banks were not directly affected by *P. papua* in the study seasons.
- Impacted: Moss banks with a moderate impact, consisting of live, beaten-up turfs neighboring the patches covered with feces and feathers. The dominant *P. strictum* moss was partially deceased in this condition. It was pink, probably due to the loss of chlorophyll. Samples were collected from moss banks 1, 4, 5.
- Desolated: A moss bank with deceased *P. strictum* and other mosses entirely covered with guano. Subsequently, it can be colonized by nitrophilic algae such as *Prasiola crispa*. Samples were collected from moss banks 1, 4, 5.

We distinguished these degrees of the penguin impact as Unaffected, Impacted and Desolated moss cover (Figure 2) and used these definitions here and after in the manuscript.

In addition to these sample types, we collected peat samples that had perished for reasons other than penguin colonization (Control Dead) as control points the margin of moss bank 3. This moss had died before the first colonies of *P. papua* appeared on the island in 2007. The primary reason for this moss death was glaciation and prolonged snow coverage in the 20th century, as discussed by Yu et al. (2016). These samples were collected to compare microbiome composition with penguin-affected and Unaffected moss.

### 2.2 Samples collection

Samples of the peat were collected by sterile spatula in 15 mL polypropylene tubes and kept at the ambient temperature during the sampling campaign (several hours) until the laboratory at Vernadsky Station, where they were immediately frozen at −80°C for further microbiological analysis. Transportation of the samples from Antarctica was facilitated on the dry ice (−30°C).

About 50 g of peat samples were collected for the chemical analysis, which was performed immediately at the Vernadsky Station.

### 2.3 Measuring the biogenic elements’ content and pH of the peat

Peat samples were homogenized and topped with distilled water in a 10:1 (H_2_O: sample) v/w ratio. Samples were mixed with water and endured for 12 h. The suspension was filtered, and the filtrate was used for further chemical analysis. рН was measured by pH-meter HI 2550 (HANNA instruments, Italy). Concentrations of mineral nitrogen (NH_4_^+^, NO_2_^+^, NO_3_^+^) and soluble phosphates in the filtrate were measured by spectrophotometer Hach Lange DR3900 (Hach, USA) in 1 inch square glass cuvettes according to the standard manufacturers’ instructions that correspond to the following methods of water analysis certified by EPA Environmental Protection Agency (USA) (Rice and Bridgewater, 2012). In brief, the concentration of NO_2_^−^ was measured by the diazotization method (reagent set NitriVer 3, Hach), the concentration of NO_3_− was measured by the cadmium reduction method (reagent set NitraVer 6, Hach), the salicylate method was used to measure NH_4_^+^ (Nitrogen-Ammonia Reagent Set, Hach), and ascorbic acid method was used to measure concentration of PO_4_^3−^ (reagent set PhosVer 3). Nitrate Standard Solution (100 mg/L), Nitrite Standard Solution (250 mg/L NO_2_^−^-N), Ammonium Standard Solution (50 mg/L), Phosphate Standard Solution (50 mg/L as PO_4_^3−^, NIST) provided by Hach (USA) were used to build the calibration curves. All measurements were performed in triplicates at 18°C.

### 2.4 DNA extraction

DNA was extracted from peat samples in duplicates by the DNeasy PowerSoil kit (Qiagen, Germany) according to the manufacturer’s instructions. DNA concentration, 260/280 and 260/230 absorbance ratio were measured by spectrophotometer Denovix DS-11 FX (Denovix, USA). In the cases where the absorbance ratios indicated heavy impurity of the sample, DNA was precipitated in 96% ethanol (in the presence of 3 M sodium acetate) and dissolved in elution buffer from a DNeasy PowerSoil kit. As a result, the DNA concentration extracted from the peat samples varied from 5.8 to 112.7 ng/μl, and the 260/280 ratio was ~1.5–1.8.

### 2.5 16S rRNA amplicon sequencing

V3-V4 regions of 16S rRNA gene were amplified with universal primers 515F (GTGCCAGCMGCCGCGGTAA) and 806R (GGACTACHVGGGTWTCTAAT) with Phusion High-Fidelity PCR Master Mix (New England, Biolabs). PCR products were checked in 2% agarose gel. Samples with 400–450 bp amplicon lengths were used for further analysis. PCR products were purified by Qiagen Gel Extraction Kit (Qiagen, Germany) and used for sequencing. Libraries were prepared using NEB Next Ultra DNA Library Pre Kit for Illumina according to the manufacturer’s instructions. Quality check of the libraries was performed by fluorimeter Qubit 2.0 (Thermo Scientific, USA) and 2,100 Bioanalyzer Instrument (Agilent Technologies, USA). A nucleotide sequence with a length of 250 bp was estimated on the NovaSeq 6000 platform.

Sequences were uploaded to the National Center for Biotechnology Information (NCBI) database under accession number PRJNA1057234.

### 2.6 qPCR analysis

Bacterial DNA extracted from peat was used to quantify the copy number of nitrogen cycle genes and 16S rRNA gene by quantitative real-time PCR. We quantified the following genes: *ureC*, *amoA*, *amoA* gene of comammox *Nitrospira* clade А, *amoA* gene of comammox *Nitrospira* clade B, *nxrB*, *nirS*, *nosZ*. The list of genes, their function and primers are presented in Supplementary Table S1.

PCR products were used as standards for each of the genes. PCR products were generated using the DNA isolated from the peat samples. The standards for each gene were agarose checked and purified with a Qiagen Gel Extraction Kit (Qiagen, Germany). The concentration of the DNA was measured by spectrophotometer Denovix DS-11 FX (Denovix, USA), and 10-fold serially diluted ranging from 1.0 × 10^−3^ to 1.0 × 10^−7^ ng/μl to be used for standard curve generation in quantitative PCR. The gene copy number per μl was calculated according to the formula:


$$\begin{array}{l} \left(\frac{X\frac{g}{\mathrm{\mu l}}DNA}{\left[\mathrm{product}\;\mathrm{length}\;\mathrm{in}\;\mathrm{base}\;\mathrm{pairs}\times 660\right]}\right)\times 6.022\times 10^{23}\\ =Y\;\mathrm{molecules}/\mathrm{\mu L} \end{array}$$

Each 25 μL PCR reaction contained the following components: 2x QuantiFast SYBR Green PCR Master Mix (Qiagen, Germany) – 12.5 μL, Primer Reverse − 2.5 μL, Primer Forward – 2.5 μL, template DNA – 1 μL, RNase-free water – 6.5 μL. The concentration of template DNA in each reaction was 20 ng/μL. The thermal conditions were different for all primers and can be found in Supplementary Table S2.

The negative control contained components for PCR reaction and RNase-free water without sample DNA. All reactions were run in triplicate on Rotor-Gene Q (Qiagen, Germany). Based on the standard curves, the threshold value (Ct) was used to determine the copy number of genes in the peat. Melting curve analysis was performed at the end of the amplification cycles to assess primer specificity and to ensure proper amplification of all target fragments.

The concentration of genes per mg of sample (wet weight) was calculated according to the formula


$$Y\;\mathrm{gene}\;\mathrm{copy}/\mathrm{mg}=\frac{\begin{array}{l} \left(\mathrm{gene}\;\mathrm{copies}\;\mathrm{per}\;\mathrm{react}i\mathrm{on}\;\mathrm{mix}\right)\\ \times \left(\mathrm{volume}\;\mathrm{of}\;DNA,\mathrm{\mu L}\right)\\ \times \left(\mathrm{dilution}\;\mathrm{factor}\right)\; \end{array}}{\left(\mathrm{sample}\;\mathrm{weight},\mathrm{mg}\right)}$$

### 2.7 Bioinformatic and statistical analysis

Sequences were grouped according to their barcodes, and barcodes and primers were trimmed. The paired ends of the sequences were joined by FLASH V1.2.7 (Magoč and Salzberg, 2011). Quality assessment and filtering were performed in QIIME V1.7.0 (Caporaso et al., 2010). Filtered data was compared to the Gold database by the UCHIME pipeline (Edgar et al., 2011). Filtered data were clustered into OTUs with a threshold of 97% in Uparse v7.0.1001 (Edgar, 2013). Each representative read was taxonomically annotated by comparison with Silva database v138.1 (Quast et al., 2013).

Data were analyzed in QIIME V1.7.0 and RStudio 4.0.4. Specifically, packages such as *vegan* (Oksanen et al., 2016), *ComplexHeatmap* (Gu et al., 2016), *ggplot2* (Wickham, 2016) and *edgeR* (Robinson et al., 2010) were used. Alpha diversity indices (OTU number, Shannon index, Faith PD index) were estimated by QIIME V1.7.0 functional and compared between groups by *t-test* (*p* < 0.05). Rarefaction analysis was performed in QIIME V1.7.0. The data on the bacterial taxa abundance and nitrogen gene number was tested for normality by the Shapiro–Wilk normality test, which revealed their non-normal distribution (*p* < 0.05). A general linear model with a negative binomial distribution (‘EdgeR’ package) was used to identify significant relationships between OTUs and intensity of the penguin impact. *p*-values were corrected to account for multiple comparisons using the false discovery rate (*q*-value) method (Benjamini and Hochberg, 1995). Results with *q* < 0.05 were considered to be significant.

A Mantel test, based on Spearman’s rank correlation, was conducted to assess whether the variance in microbial composition is explained by the pH of the substrate, utilizing distance matrices calculated using the Bray–Curtis measure. ANOSIM tests, based on Bray–Curtis distance matrices, were applied to assess whether the variabilities between microbial communities are explained by the intensity of ornitogenic impact or the sampling place (moss bank). A Non-metric Multidimensional Scaling (NMDS) plot of the OTU abundance was built on the Bray-Curtis distance matrix. Welch *t-test* was applied to compare the chemical parameters of the different types of peat. Kruskal-Wallis and Dunn tests were used to compare the nitrogen-cycle gene numbers in different types of peat. Spearman’s rank correlation coefficient was calculated to reveal a correlation between nitrogen-cycling genes and the relative abundance of bacterial taxa.

## 3 Results

### 3.1 Chemical parameters of the peat

All the samples were acidic except the Desolated peat. The Unaffected and Impacted peat had acidic pH (4.7 ± 0.05 and 5.4 ± 0.4), though Welch’s two-sample *t-test* revealed a significant difference between these two groups of samples (*T* = −4.45, *p* < 0.05, *df* = 15.0). pH of the Desolated peat significantly differed from the Unaffected peat (*T* = 5.8, *p* < 0.05, *df* = 14.4) and comprised 7.2 ± 0.6. The moss bank affected by factors other than penguin colonization was similar to the Unaffected peat from this study (4.6 ± 0.03).

**Figure 3:**
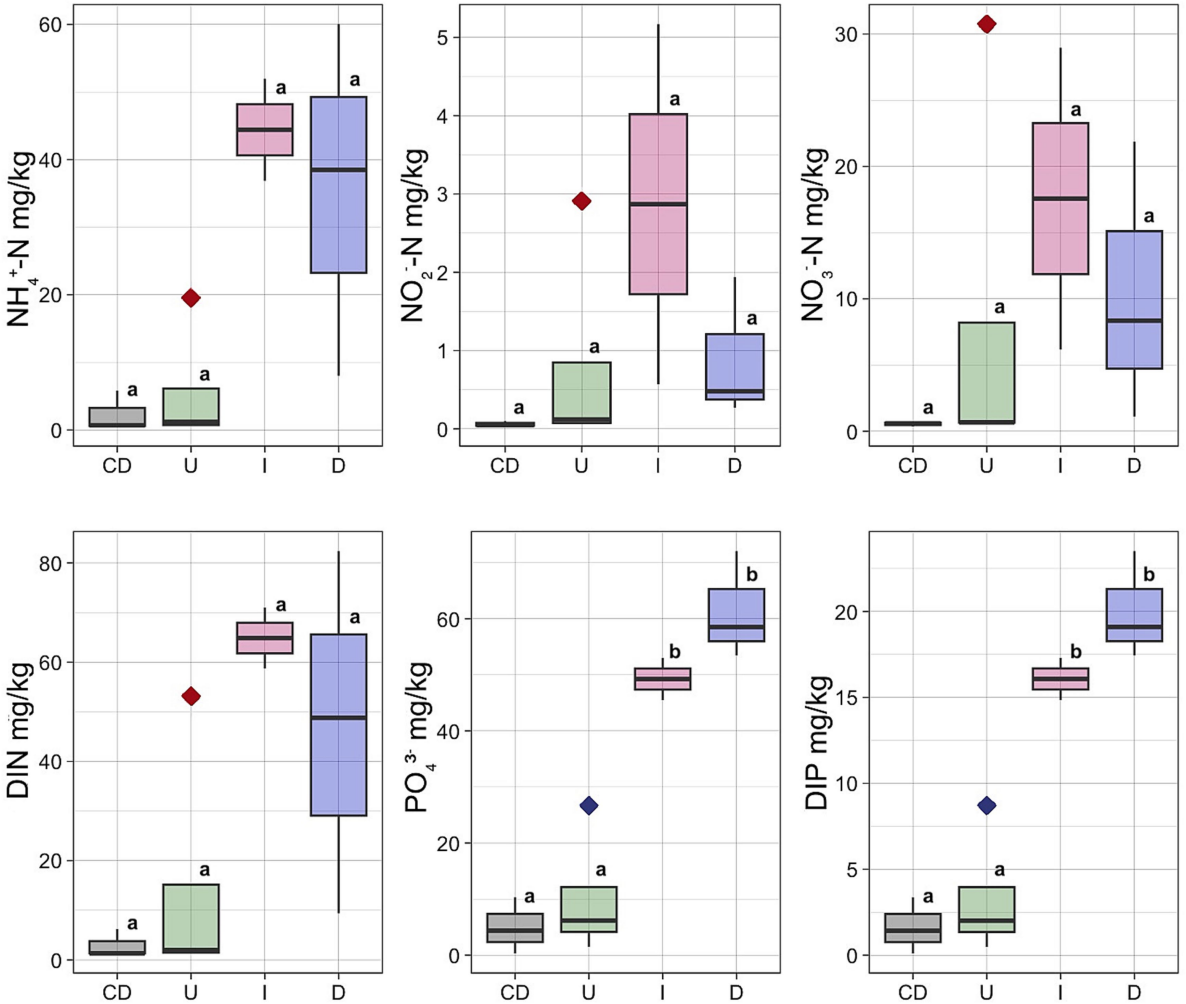
Concentrations of the NH_4_^+^, NO_2_^−^, NO_3_^−^, PO_4_^3^−, DIN and DIP in different types of the peat: U, Unaffected; I, Impacted; D, Desolated; CD, Control Dead; Outlier marked by red diamond – moss bank 5; Outlier marked by blue diamond – moss bank 1.

Concentrations of the NH_4_^+^, NO_2_^−^, NO_3_^−^ and content of dissolved inorganic nitrogen (DIN) had the lowest concentrations in the Unaffected peat and Control Dead peat (Figure 3). The concentration of all these nutrients increased in the Impacted peat. On the contrary, the content of nitrogen compounds tended to decrease in the destroyed substrate. In particular, the concentration of NH_4_^+^ fell from 44.4 ± 10.6 mg/kg in Impacted peat to 35.5 ± 26.2 mg/kg in Desolated peat. Similarly, the concentration of NO_2_^−^ and NO_3_^−^ decreased from 2.9 ± 3.2 and 17.6 ± 16.1 mg/kg to 0.9 ± 0.9 and 10.4 ± 10.5 mg/kg, respectively.

The concentration of PO_4_^3−^ and dissolved inorganic phosphorus (DIP) increased when ornithogenic impact grew with the highest estimates in the Desolated peat samples (Figure 3). The concentration of PO_4_^3−^ increased from 49.3 ± 9.6 mg/kg in the Impacted peat to 61.4 ± 5.6 mg/kg in the Desolated peat. In contrast, it was 5.0 ± 2.9 mg/kg in the Control Dead samples. Similarly, the concentration of DIP increased from 16.1 ± 3.2 to 20.0 ± 1.8 mg/kg in the Impacted and Desolated peat. Control Dead peat samples contained lower DIP concentrations, numbering 1.6 ± 0.9 mg/kg.

The Unaffected peat sample group included an outlier taken from the moss bank 5, exhibiting elevated concentrations of all nitrogen compounds (Figure 3). NH_4_^+^ was 19 times higher than the average of the remaining Unaffected samples, NO_2_^−^ was higher by 29 times, and NO_3_^−^ was higher by 46 times. The total DIN exceeded the average values for the Unaffected samples by 29 times. An outlier within the Unaffected peat group also exhibited an elevated concentration of soluble phosphorus, represented by a sample collected from the moss bank 1 (Figure 3).

### 3.2 16S rRNA amplicon sequencing output

Illumina NovaSeq 6,000 identified between 6,032 to 126,755 reads of the partial 16S rRNA gene per sample, with an average length of 414 ± 5 bp. Based on rarefaction curves, significantly higher estimates would not be achievable by deeper sequencing (Supplementary Figure S1). After quality filtering and clustering (threshold 97%) of the sequencing data, we obtained from 813 to 1990 OTUs per sample. Averages of the OTU number and diversity indices are presented in Table 1.

**Table 1:** Diversity indices of the microbial communities of the peat (average ± standard deviation).

	OTU number	Shannon, H	Faith, PD
Unaffected	1,276 ± 242	7.1 ± 0.9	129 ± 14
Impacted	1,564 ± 378	7.7 ± 0.6	143 ± 46
Desolated	1,185 ± 255	5.6 ± 1.5	117 ± 22
Control Dead	1,302 ± 187	7.5 ± 0.3	122 ± 18

The microbial communities in Unaffected and Control Dead samples exhibited comparable OTU numbers and diversity indices. In contrast, the Impacted peat microbiomes showed slightly elevated OTU, Shannon, and Faith values compared to the other microbial communities. Conversely, the Desolated peat microbial communities displayed lower diversity than those in the other three groups. Notably, a significant difference was only observed for the Shannon index between Impacted and Desolated peat (*t* = −2.9, *df* = 5.4, *p* < 0.05), indicating a distinct diversity pattern in these two peat types.

### 3.3 Taxonomic composition of moss banks’ microbial communities

**Figure 4:**
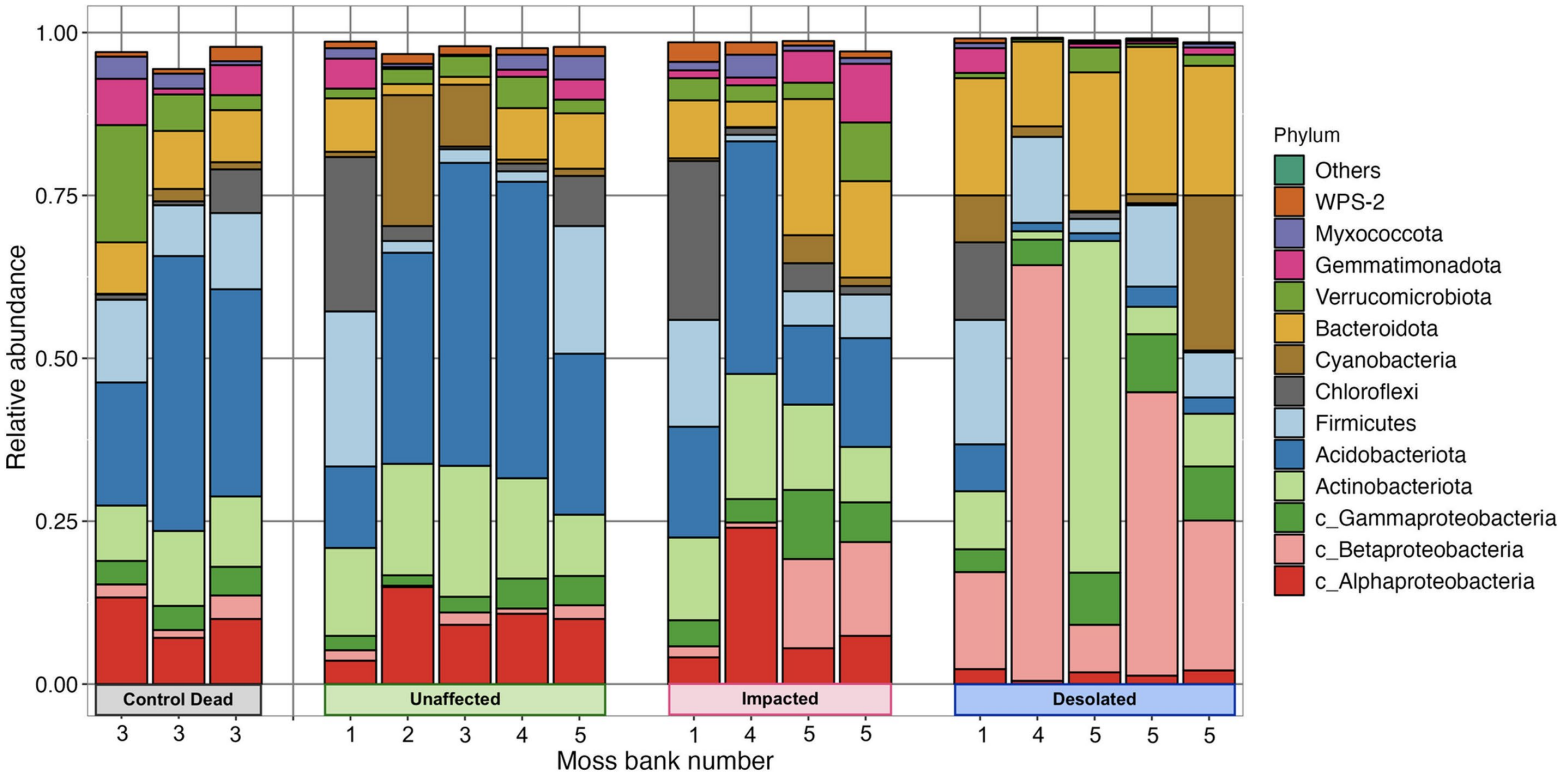
Abundance of bacterial phyla in different types of peat and different moss banks.

*Proteobacteria*, *Actinobacteria*, *Acidobacteria*, *Firmicutes*, *Chloroflexi*, *Cyanobacteria*, and *Bacterioidota* were identified as the most abundant phyla in the peat of moss banks affected by penguins’ colonization (Figure 4). *Proteobacteria* dominated across all conditions, constituting 14.1 ± 3.9% in Unaffected, 23.9 ± 9.4% in Impacted, 38.6 ± 6.0% in Desolated, and 16.3 ± 3.8% in Control Dead peat. *Actinobacteria* exhibited variations with percentages of 15.1 ± 4.0%, 13.4 ± 4.4%, 14.7 ± 20.5%, and 10.3 ± 1.6% in the respective conditions. *Acidobacteria* represented 32.3 ± 14.4% in Unaffected, 20.4 ± 10.5% in Impacted, 3.1 ± 2.4% in Desolated, and 31.0 ± 11.6% in Control Dead peat. *Firmicutes* (9.7 ± 8.6%) and *Chloroflexi* (5.2 ± 7.8%) maintained consistent distributions across conditions. *Cyanobacteria* comprised 6.4 ± 8.5% in Unaffected, 1.5 ± 1.9% in Impacted, 6.8 ± 9.8% in Desolated, and 1.0 ± 0.9% in Control Dead peat, while *Bacterioidota* ratios were 5.5 ± 3.7% in Unaffected, 12.1 ± 7.4% in Impacted, 18.9 ± 3.7% in Desolated, and 8.3 ± 0.6% in Control Dead peat.

The ratio of some bacterial phyla, such as *Betaproteobacteria* and *Bacterioidota*, differed when the ornithogenic impact on the moss increased. The relative abundance of these phyla increased from 1.3 ± 0.8% and 5.5 ± 3.7% in the Unaffected peat to 30.5 ± 23.0% and 19.0 ± 3.7% in the Desolated peat. On the other hand, the relative abundance of *Acidobacteria* decreased from 32.2 ± 14.4% in Unaffected peat to 3.1 ± 2.4%.

**Figure 5:**
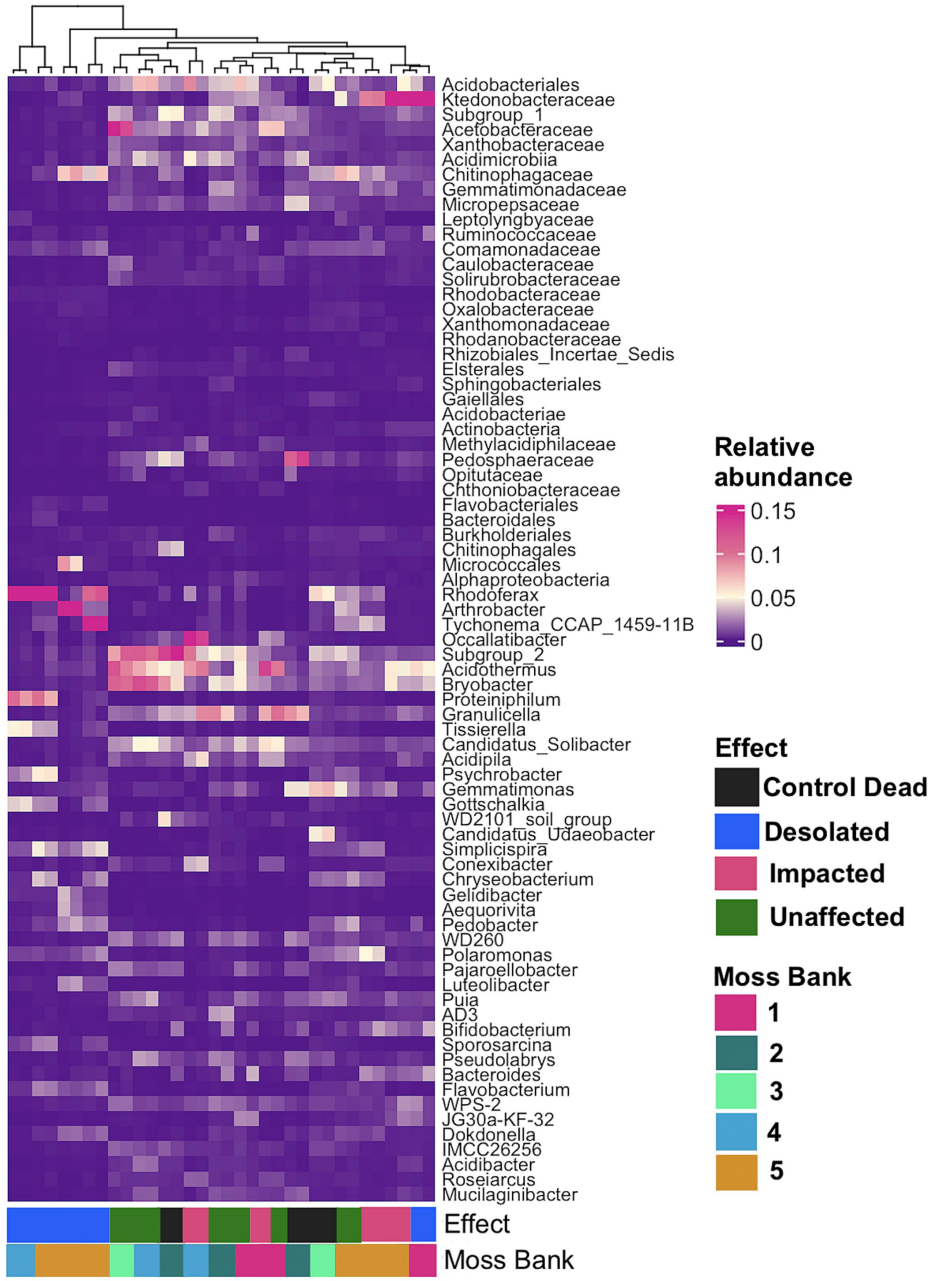
The relative abundance of bacterial taxa at the lowest taxonomic level, identified as differentially abundant OTUs (*q* < 0.05) across various types of peat samples using a general linear model (GLM) with a negative binomial distribution.

A general linear model (GLM) with a negative binomial distribution identified OTUs with significantly differential abundance (*q* < 0.05) across Unaffected, Impacted, Desolated, and Control Dead peat samples (Supplementary Table S3). The heatmap in Figure 5 illustrates the relative abundance of taxa, the OTUs of which were identified as differentially abundant across various types of peat samples (*q* < 0.05).

Figure 5 showcases a distinctive microbial assemblage predominantly found in Unaffected moss:

- Acidobacteria: *Subgroup 1*, *Subgroup 2*, *Bryobacter*, *Granulicella*, *Occalatibacter*, *Candidatus Solibacter*, *Acidimicrobia*, *Acidobacteriaceae*, *Acidobacteriales*;
- Actinobacteria: *Acidothermus*, *Conexibacter*, IMCC26256, S*olirubrobacteraceae*;
- Alphaproteobacteria: *Pseudolabrys, Roseiarcus, Xanthobacteraceae, Caulobacteraceae, Acetobacteraceae, Micropepsaceae*, *Elsterales;*
- Bacteroidota: *Puia*, *Mucilaginibacter*;
- Chloroflexi: AD3, JG30a-KF-32, *Ktedonobacteraceae*;
- Gammaproteobacteria: WD260, *Acidibacter*;
- Gemmatimonadota: *Gemmatimonadaceae*;
- Myxococcota: *Pajaroellobacter*;
- Verrucomicrobia: *Pedosphaeraceae*, *Methylacidiphilaceae*, *Opitutaceae*;
- WPS-2: WPS-2.

Genetic markers of these taxa were abundant in the Control Dead peat, suggesting that this microbial assemblage may represent an initial community for moss bank peat. These microbial taxa are present in Impacted moss, although they may be dormant or deceased in this case.

In contrast, the Impacted moss harbors a secondary microbial group alongside the initial assemblage. This secondary group comprises taxa such as:

- Actinobacteria: *Arthrobacter*;
- Bacteroidota: *Tissierella*, *Pedobacter*;
- Betaproteobacteria: *Simplicispira*, *Polaromonas*;
- Cyanobacteria: *Tychonema*;
- Firmicutes: *Gottschalkia*, *Sporosarcina*;
- Flavobacteria: *Chryseobacterium*, *Gelidibacter*, *Flavobacterium*, *Aequorivita*;
- Gammaproteobacteria: *Psychrobacter*, *Luteoibacter*;
- Verrucomicrobia: *Candidatus Udeobacter*.

This second microbial group becomes a core component in the Desolated mosses, where, unlike the Impacted mosses, the initial peat microbiota is nearly absent.

ANOSIM statistics revealed moderate correlation of the microbimes composition with the intensity of the penguins’ impact (*R* = 0.4, *p* < 0.05) and moss bank (*R* = 0.4, *p* < 0.05). According to the Mantel test results based on Spearman correlation, there is strong correlation between the microbiome composition and pH of the substrate (*R* = 0.65, *p* < 0.05).

**Figure 6:**
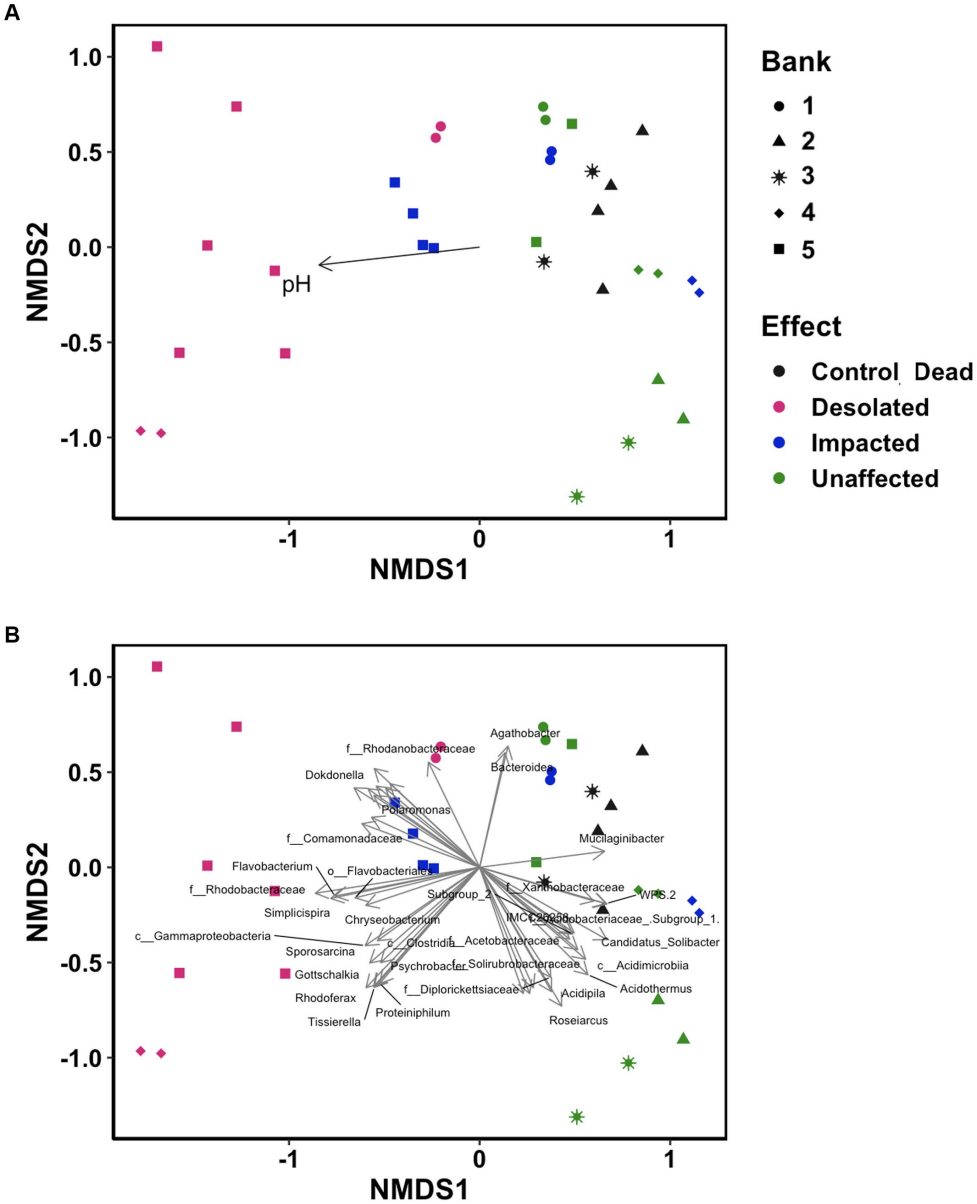
Non-metric multidimensional scaling (NMDS) ordination analysis on the distinctive distribution of OTU build on the Bray–Curtis dissimilarity matrix. Data points are color-coded by ornithogenic impact and symbolized by moss bank. **(A)** pH as intrinsic vector driving the distribution pattern (*p* < 0.05); **(B)** microbial taxa (*p* < 0.001) driving the distribution pattern.

Non-metric multidimensional scaling (NMDS) ordination analysis was conducted using OTUs to visualize differences in microbiomes (Figure 6). The microbiomes of Desolated moss formed a distinct cluster, in contrast to the Unaffected moss. The microbial communities of Impacted moss represented a transitional state between the Unaffected and Desolated mosses’ microbiomes. pH played a significant role (*p* < 0.05) in distinguishing microbial communities, as depicted in Figure 6A. Figure 6B illustrates the taxa (*p* < 0.001) that may contribute to the differences between microbiomes in different types of peat.

### 3.4 Changes in the microbial communities functioning

**Figure 7:**
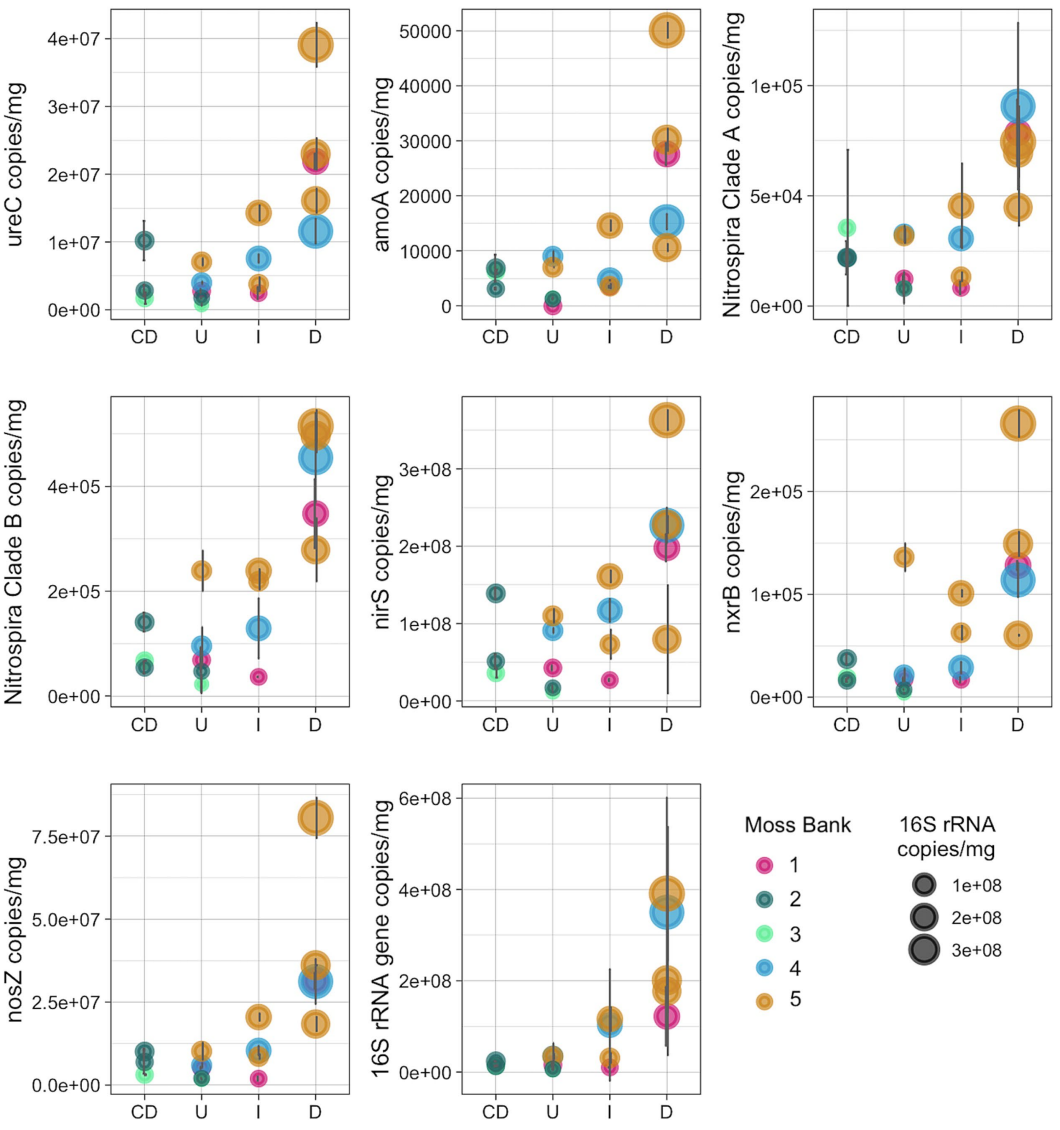
Number of copies of the nitrogen-cycle and 16S rRNA genes per mg of peat (wet weight): U, Unaffected; I, Impacted; D, Desolated; CD, Control dead.

The copy number of 16S rRNA gene and several genes related to the nitrogen cycle were quantified, such as *ureC*, bacterial *amoA*, *amoA* of the commamox *Nitrospira* clade A- and B, *nxrB*, *nirS* and *nosZ* (Figure 7; Supplementary Table S4).

The nitrogen cycle gene copy number increased in the Impacted and Desolated moss (Figure 7). The Kruskal-Wallis rank sum test supports the differential copy number of the *ureC*, *amoA*, commamox *amoA* of *Nitrospira* clades A and B, *nosZ* genes among the groups tested (*p* < 0.05). Dunn test revealed a significant difference (*p* < 0.05) in the abundance of all nitrogen cycle genes except for *nxrB* between the Unaffected and Desolated peat. The detailed *p-values* of the Dunn test are presented in the Supplementary Table S4. Differences in the abundance of the genes between the other groups of samples (as Unaffected and Impacted, Impacted and Desolated) were not significant according to the Dunn test.

The copy number of the 16S rRNA gene increased when the impact of the penguins on the moss banks intensified (*p* < 0.05). Unlike the Desolated moss banks, the copy number of nitrogen cycle genes and 16S rRNA genes in Control Dead samples remained comparable to the Unaffected samples.

**Figure 8:**
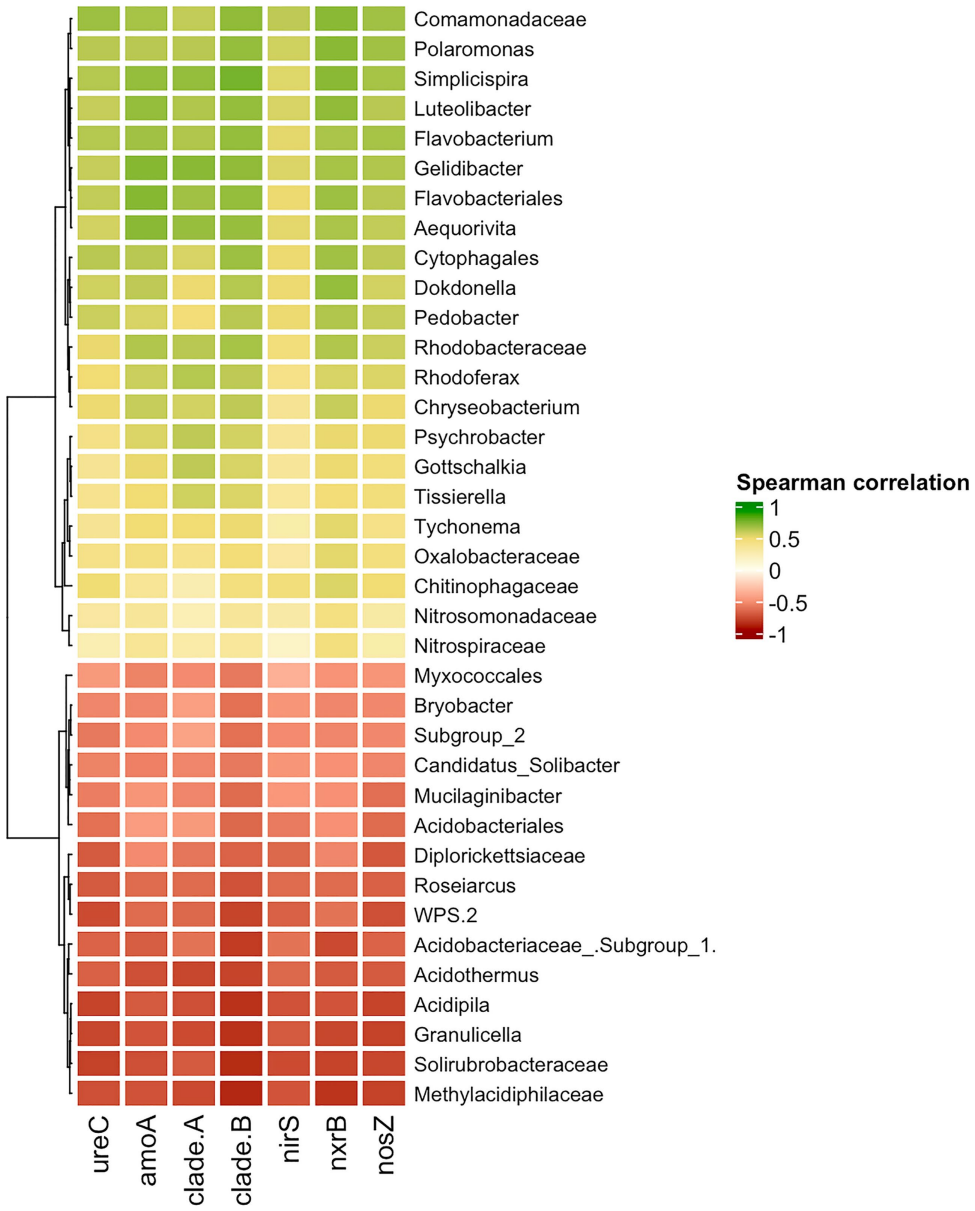
Spearman correlation between nitrogen-cycle genes and bacterial taxa. Only moderate (|0.5|) and strong (|0.5–1.0|) correlation is shown.

The Spearman correlation was calculated to uncover what taxa are likely involved in the enrichment of the nitrogen-cycling genes. The group of taxa that had a negative correlation with the nitrogen cycling genes was native to Unaffected moss bank samples (Figure 8), which is likely the result of eliminating these bacteria.

A positive correlation between the abundance of taxa and the presence of nitrogen-cycling genes may suggest that enrichment with specific taxa could lead to enrichment in genes associated with the nitrogen cycle. Nevertheless, interpreting the results should be cautiously approached, given the challenge of distinguishing between causation and spurious correlation in this particular case. The comammox *amoA* gene exclusively belongs to *Nitrospira* members. Therefore, any correlation observed between comammox *amoA* of *Nitrospira* clades A and B with *Polaromonas*, *Simplispira*, *Rhodoferax*, or other taxa is more likely to be a coincidence. The other nitrogen cycle genes examined in the study belong to diverse taxonomical groups and may enhance the functional capabilities of various microorganisms.

## 4 Discussion

This study explores the influence of gentoo penguin colonization as an indirect impact of climate change on alterations in terrestrial ecosystems in the maritime Antarctic, specifically focusing on microbial communities. Our analysis centers on the changes in microbiota within the peat formed by the Tall moss turf subformation, triggered by the rapid establishment of gentoo penguin colonies on Galindez Island (Argentine Islands) and Cape Tuxen on the mainland of Kyiv Peninsula. It encompasses the examination of the chemical composition of the peat, the taxonomic composition of microbiomes, and the abundance of nitrogen-cycle gene copy numbers in peat samples collected from sites differently affected by penguin presence.

### 4.1 Chemical composition of the peat

The impact of *P. papua* colonization on moss banks induces alterations in the chemical composition of the peat substrate. Notably, penguin colonization leads to an elevation in peat pH and an increase in the concentration of soluble nitrogen and phosphorous compounds. Our results for Unaffected peat, except for a few points, are consistent with the previous estimates by Parnikoza et al. (2017), in which histozols formed by *Polytrichum strictum* showed low phosphorus content, indicating a low influx of zoogenic materials. Unaffected peat from moss bank 5 had an elevated content of nitrogen compounds. It is possible that, due to the moss bank’s location on a steep slope, ornithogenic organics may have leached from the penguin colonies situated above. Alternatively, the volatile ammonium from the penguin rookery could be transported (Lindeboom, 1984). The latter scenario might even promote vegetation growth (Crittenden et al., 2015; Riddick et al., 2016). Thus, the moss bank could be enriched with biogenic compounds without displaying signs of negative eutrophication impact. Similarly, the increased phosphorus content was detected in the Unaffected peat of the moss bank 1. We might speculate that ornithogenic impact in this location occurred earlier, preceding the study, as indicated by the accumulation of biogenic compounds without desolating the moss carpet. Unlike nitrogen compounds, phosphorus does not evaporate from the environment, allowing us to observe its accumulation.

Penguin guano, comprising 15–30% nitrogen and 10% phosphorus (Lorrain et al., 2017), is a key contributor to the changes in the chemical composition of peat. Excess nitrogen and phosphorus originate from the adjacent water regions of the Southern Ocean, where gentoo penguins primarily forage for krill (*Euphausia superba*). As this specific source of nitrogen and phosphorus becomes predominant in the altered ecosystem of moss banks, it can be speculated that a transformation is occurring in the primary terrestrial polar ecosystem. In this original ecosystem, the atmosphere and bedrock can be assumed as the main nutrient sources. However, the ongoing transformation leads to a new ecosystem that relies on the sea as its primary nutritional source.

The rise in pH from acidic to alkaline is linked to the availability of ammonia in the feces and concurrent biochemical processes within the substrate. The total nitrogen in penguin feces is primarily composed of uric acid, followed by proteins, ammonium, and nitrates (Lindeboom, 1984). Uric acid and proteins undergo degradation into ammonia nitrogen, contributing to substrate alkalization and subsequent ammonia volatilization from the rookery (Lindeboom, 1984). The role of uric acid conversion to ammonium was demonstrated by an increase in the pH of the ornithogenic soil under the penguin colony from 6.7–6.9 in January to 7.1–9.0 in March (Grzesiak et al., 2020).

The elevated pH facilitates ammonia volatilization, thereby explaining the decrease in the concentration of soluble nitrogen compounds in Desolated moss banks compared to Impacted ones. Unlike nitrogen, phosphorus does not evaporate from the rookery, elucidating the gradual accumulation of phosphate (PO₄^−^) and inorganic phosphorus (PIN) in Impacted and Desolated peat environments.

### 4.2 The taxonomic composition of the moss banks differently affected by the penguins’ colonization

The initial microbiota of the peat was primarily dominated by *Acidobacteria*, followed by *Actinobacteria* and *Alphaproteobacteria*. In contrast, microbial communities associated with other bryophytes exhibited distinct compositions. For instance, in peat formed by *Sphagnum* mosses, *Proteobacteria* (56.4%) and *Acidobacteria* (8.2%) were dominant (Kolton et al., 2022). On the Keller Peninsula (King George Island), the moss carpet formed by *Saniona* sp. showed *Actinobacteria* (25%), *Proteobacteria* (20%), and *Bacteroidetes* (18%) as the most abundant phyla (Câmara et al., 2021). Unfortunately, we found no available data on the microbial composition of moss banks formed by *P. strictum*. Hence, our findings likely represent the first report on the microbial communities’ composition in Unaffected peat formed by Tall moss turf subformation.

The chemical composition of peat and penguin feces is quite contrasting, leading to the elimination and replacement of the indigenous peat microbiota. The increase in pH was a contributing factor to the elimination of initial peat microorganisms, which typically thrive in acidic conditions. In particular, initial microbial taxa found in Unaffected peat, such as *Acidobacteria* members, disappear with increasing intensity of penguin influence. The taxonomic composition of soil microbial communities from Fildes Peninsula, King George Island, with different intensities of penguins’ influence, was estimated by Guo et al. (2018). Still, no such variation in the composition of microbial communities was detected. The soil had a higher pH (5.5–6.25) and a different microbial composition of the initial microbial communities, so penguin feces input was less environmentally disruptive.

Colonization of the moss bank by penguins results in the introduction of new microorganisms into the peat. Notably, there was a pronounced increase in the abundance of *Betaproteobacteria* and *Bacteroidota*. Interestingly, the composition of ornithogenic soil on Fildes Island differed from the impacted peat, with *Proteobacteria* (33.4%), *Actinobacteria* (19.9%), and *Gemmatimonadetes* (15.2%) dominating the microbial community (Guo et al., 2018).

The group of bacteria that emerged in the Impacted and Desolated peat and their ability to proliferate and function in the field environment of Antarctica is of particular interest. This group includes taxa associated with birds’ gut. *Gottschalkia* is usually found in the intestines and guano of birds. For instance, a high abundance of the *Gottschalkiaceae* family was found in *P. adeliae* guano samples (Grzesiak et al., 2020). *Tissierella* and *Flavobacterium*, identified in the Impacted peat of Galindez Island and Tuxen, were previously detected in the guano of *P. adeliae* (Grzesiak et al., 2020). Members of the *Psychrobacter* genus occur in animal microbiomes, including the respiratory tract of marine mammals (Apprill et al., 2017), the skin of marine mammals (Apprill et al., 2014), the throat and gut of birds (Kämpfer et al., 2015, 2020). *Chryseobacterium* is frequently linked to the intestines of animals, including penguins; proteolytic *Chryseobacterium* sp. was identified in the guano of *P. adeliae* (Grzesiak et al., 2020). *Fusobacteriaceae*, *Moraxellaceae*, *Leuconostocaceae*, *Lachnospiraceae*, *Streptococcaceae*, *Campylobacteriaceae*, *Porphyromonadaceae*, and *Helicobacteriaceae* (Dewar et al., 2013), as well as *Carnobacteriaceae*, *Eurysipelotrichaceae*, *Moraxellaceae*, and *Pseudomonadaceae*, were identified within the penguins’ intestinal microbiota. However, they were not detected in the peat in our study.

The environmental conditions in Antarctica markedly differ from those within the penguins’ intestines, posing a challenge for the assimilation of such microbiota. The presence of 16S rRNA sequences from these taxa in the metagenome does not necessarily indicate the activity of these microorganisms, as genetic markers can also originate from deceased or dormant organisms. The persistence of genetic markers may be attributed to the continual influx of guano microbiota or the potential adaptation of certain representatives to their new environmental conditions.

In this context, examining moss bank 1 becomes valuable—birds initially colonized it but have since abandoned it, eliminating the fresh influx of guano. Moss bank 1 is an illustrative example of system adaptation and recovery following a significant disturbance. Notably, *Gottschalkia*, *Tissierella*, and *Psychrobacter* were absent in Desolated moss bank 1 despite their presence in other Desolated mosses. In contrast, *Chryseobacterium* persisted in the environment. *Simplicispira*, *Polarobacter*, and *Tychomena*, which were abundant in other Desolated moss bank samples, also exhibited persistence. Notably, some of these, such as *Tychomena*, are not associated with bird gut microbiota; nevertheless, they all benefit from environmental changes.

Previous studies have focused on comparing the microbial composition of abandoned penguin sites and actively colonized soils (Aislabie et al., 2009; Kim et al., 2012). However, our results differ both from these studies. These variations may be attributed to the distinct conditions of the studied sites, including locations in the Ross Sea region, the North and central part of the WAP. In particular, according to Aislabie et al. (2009), formerly penguin-colonized sites at Cape Hallett, around the Ross Sea region of Antarctica, contained *Actinobacteria* and *Xanthomonas*. In actively penguin-colonized soils, the dominant taxa included *Firmicutes* and *Psychrobacter. Ferruginibacter*, *Sulfuritalea*, *Polaromonas*, and *Rhodanobacter* dominated the soil of the abandoned penguin sites on the southern coast of the Barton Peninsula of King George Island (Kim et al., 2012). *Thermohalobacter*, *Tissierella*, *Carnobacteriaceae*, *Desulfonispora, Psychrobacter* and *Pseudomonas* were found in high abundance dominant in actively penguin-colonized soils (Kim et al., 2012).

Santamans et al. (2017) and Grzesiak et al. (2020) have addressed intestinal microbiota survival challenges in Antarctic field conditions. The resilience of such microbiota in the harsh conditions of Antarctic terrestrial biotopes may be attributed to the formation of anaerobic microniches, a result of the accumulation of excessive organic matter, trampling, and the consequent high biological oxygen consumption in the environment (Santamans et al., 2017). Grzesiak et al. (2020) discussed modifying environmental conditions over time and adapting birds’ intestinal microbiota to these conditions. The guano microbiome of Adélie penguins, sampled at their nesting sites during the summer season, predominantly consisted of dead or inactive bacteria (Grzesiak et al., 2020). Conversely, guano sampled at the end of the summer season contained ten times more bacterial cells and psychrophilic enzymes. Most bacteria in the latter sample exhibited intact membranes, suggesting their adaptation to environmental conditions. The increased bacterial count in fermented guano indicated proliferation, adaptation, and the formation of new niches.

### 4.3 Effect of the penguins’ colonization on the functioning of peat microbial communities

A peat’s chemical and microbial composition shift inevitably impacts microbial communities’ functionality. We quantified the copy number of several genes involved in various stages of the nitrogen cycle. These genes collectively regulate urea conversion, the primary component of penguin feces, into ammonia, nitrite oxide, and nitrate oxide. Across all studied genes related to the nitrogen cycle, there was an increase in copy number in Impacted peat, reaching its peak in Desolated peat. The uneven distribution of these genes in the peat, influenced differentially by ornithogenic impact, underscored alterations in the functional activity of microbial communities. An increase in the copy number of nitrogen cycle genes is logically related to the high input of nitrogen compounds into the environment and the enrichment of the microbiome with taxa containing such genes. On the other hand, according to Björck et al. (1991), nitrogen loss through denitrification or leaching from moss banks was insufficient. It’s noteworthy that in Desolated moss, the copy number of nitrogen cycle genes was the highest, despite the concentration of soluble nitrogen compounds being lower compared to Impacted moss. This fact might suggest elevated activity among nitrogen cycling bacteria, indicating their adaptation to the continuous impact of nitrogen compounds and change in the environmental conditions.

*Polaromonas*, *Simplisiprira*, *Dokdonella*, *Pedobacter*, *Psychrobacter*, *Rhodoferax,* etc., revealed a strong significant correlation to *ureC* and *nosZ* gene copy number, indicating their potential involvement in urea decomposition and denitrification processes. A moderate and significant correlation was observed between *Nitrosomonadaceae* abundance and *amoA* copy number, and as well as between *Nitrospiraceae* and *nrxB* gene copy number. While an increase in *amoA* and *nrxB* gene copy numbers due to penguin colonization pressure could be associated with *Nitrosomonadaceae* and *Nitrospiraceae* proliferation, no significant elevation of these bacteria ratio was observed in the Impacted and Desolated peat samples.

According to the literature, certain taxa detected in penguin-affected environments can break down uric acid, a major component of guano, and contribute to denitrification. Specifically, members of *Gottschalkia* have demonstrated the capability to metabolize uric acid as their sole source of carbon and energy (Poehlein et al., 2017). Additionally, some *Psychrobacter* members possess lipolytic properties and the capacity to degrade uric acid (Grzesiak et al., 2020). In a study focusing on microbial communities and genes involved in N_2_O emissions in Antarctic soils impacted by marine animals, Ramírez-Fernández et al. (2021) found that the only metagenome-assembled genome (MAG) related to *Nitrosospira lacus* included an ammonia monooxygenase (*amoA*) gene and comprised the complete nitrification pathway. *Rhodoferax* is known to thrive using Fe(III) and NO₃^2−^ as electron acceptors, with organic acids serving as electron donors (Finneran et al., 2003). In guano-enriched peat microbial communities, these bacteria may contribute to nitrate reduction. The genus *Simplicispira* enriched in the Impacted and Desolated peat encompasses aerobic and facultative anaerobic bacteria and has denitrification capabilities (Siddiqi et al., 2020), According to Ramírez-Fernández et al. (2021), only three MAGs found among 100 MAGs obtained from animal-impacted soils included all necessary genes for the complete denitrification pathway (i.e., reductions from NO_3_^−^ to N_2_): *Pandoraea thiooxydans*, *Pseudomonas stutzeri* and *Oblitimonas alkaliphila*. On the contrary, most MAGs belonging to diverse bacterial phyla carried an incomplete denitrification pathway (Ramírez-Fernández et al., 2021). That shows that denitrification is a highly abundant process carried out by the diverse microbial taxa in Antarctic animal impacted soils.

## 5 Conclusion

One of the consequences of climate change indirect effects in the maritime Antarctic region is the alteration of terrestrial vegetation, marked by the elimination of moss banks and the associated acidophilic microbial community. This change is attributed to the expansion of nesting territories by gentoo penguins. The proliferation of microorganisms introduced with penguin guano is a contentious issue requiring further research. Nevertheless, it is evident that conditions conducive to the functioning of a new microbial community, shaped by the chemical and microbial composition of penguin guano, have been established. The functional capacity of this microbial community differed from the initial peat community, as demonstrated in this study with the example of the nitrogen cycle. Likely, functional activities related to carbon will also vary, potentially leading to more intensive mineralization of organic compounds previously conserved in the form of peat. Addressing this question would require future attention through functional assays.

## Data availability statement

The dataset of the16S rRNA amplicon metagenomic sequences generated for this study can be found in National Center for Biotechnology Information (NCBI) database (https://www.ncbi.nlm.nih.gov/) under accession number PRJNA1057234.

## Ethics statement

Written informed consent was obtained from the individual(s) for the publication of any potentially identifiable images or data included in this article.

## Author contributions

YP-K: Formal analysis, Investigation, Methodology, Visualization, Writing – original draft, Writing – review & editing. IP: Conceptualization, Writing – review & editing. AY: Investigation, Writing – review & editing. OS: Investigation, Writing – review & editing. MP: Visualization, Writing – review & editing. MD: Investigation, Writing – review & editing. OY: Investigation, Writing – review & editing. ED: Project administration, Resources, Writing – review & editing.
