# Supplementary material for: From acidophilic to ornithogenic: microbial community dynamics in moss banks altered by gentoo penguins
Source: Front Microbiol. 2024 Mar 7;15:1362975. doi: 10.3389/fmicb.2024.1362975 (PMC10959021; doi:10.3389/fmicb.2024.1362975)
Supplement: Supplementary file 1 [file Data_Sheet_1.pdf]

Supplementary Material

Supplementary Figure

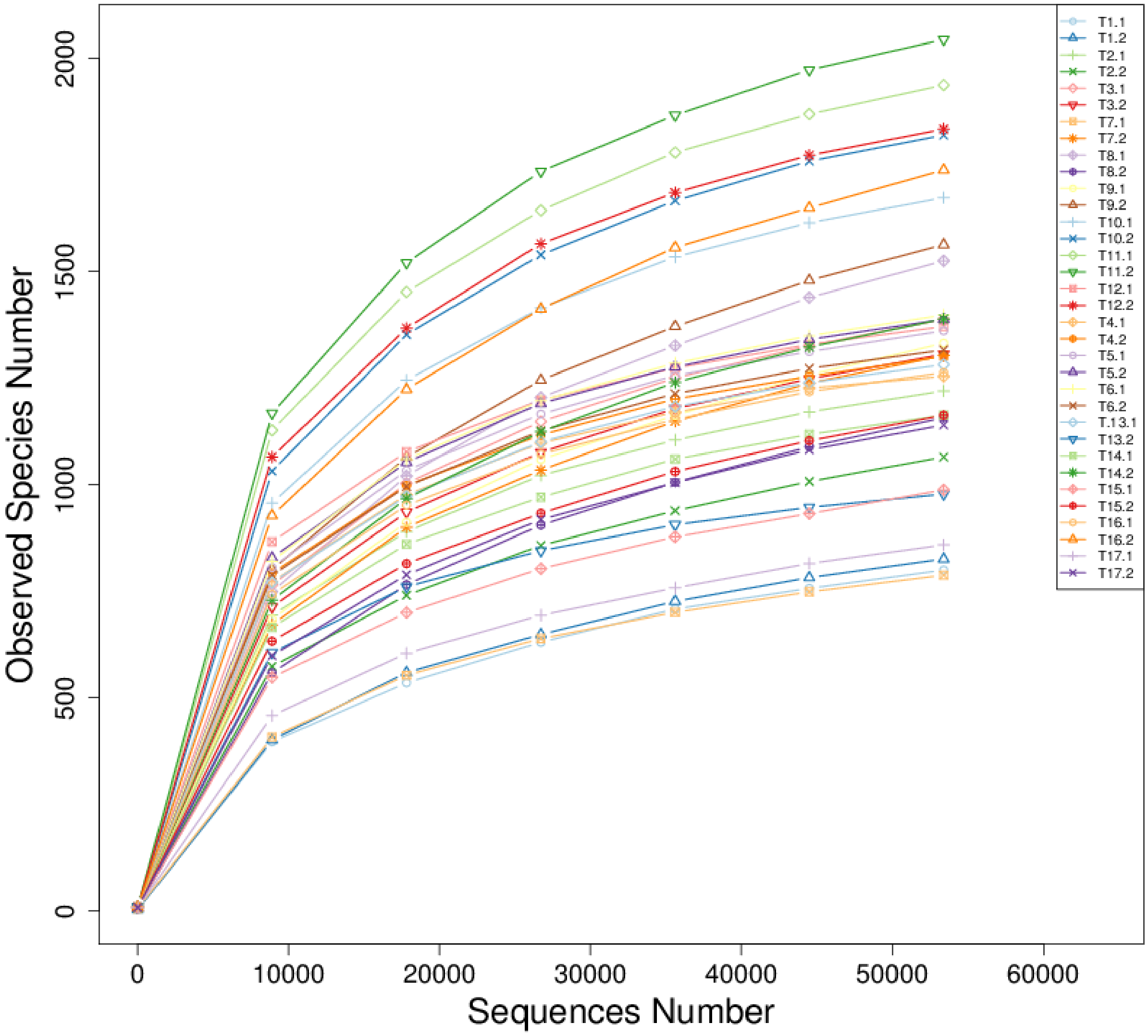

Supplementary Figure 1 - Rarefaction curves of partial sequences of bacterial 16S rRNA genes from differentially affected moss banks: Unaffected moss - T.3.1, T.3.2, T.6.1, T.6.2, T.12.1, T.12.2, T.15.1, T15.2, T.17.1, T.17.2; Impacted moss - T.2.1, T.2.2, T.5.1, T.5.2, T.10.1, T.10.2, T.11.1, T.11.2; Desolated moss - T.1.1, T.1.2, T.4.1, T.4.2, T.7.1, T.7.2, T.8.1, T.8.2, T.9.1, T.9.2; Control Dead - T.13.1, T.13.2, T.14.1, T.14.2, T.16.1, T.16.2

Supplementary Material

1. Supplementary Tables

Table S1 - Primers, product sizes (**Pr, bp**) and annealing temperatures (AT, °C) of the nitrogen-cycling genes involved in the study

**Gene**	**Primer**	**Sequence**	**Pr, bp**	**AT, °C**	**Function**	**Reference**
ureC	L2F_V1	CGGCA AGGCCGGCAACCC	386	50	Urease synthesis	Oshiki *et al.*, 2018
	733R	GTBGHDCCCCAR TCYTCRT				
amoA	amoAF	TGGCTCGTGACAGCGT TAAT	95	60	Bacterial ammonifi-cation	Khangembam, Sharma and Chakrabarti, 2017
	amoAR	ACGATTGGCAAGTGG GTCG				
amoA	C576r	GAAGCCCATRTARTCN GCC	199	55	Gene of comammox *Nitrospira* clade B (Jiang *et al.*, 2020)	Jiang *et al.*, 2020
	CB377f	GTACTGGTGGGCBAAY TT				
amoA	CA377f	GTGGTGGTGGTCBAAY TA	199	55	Gene of comammox *Nitrospira* clade A	Jiang *et al.*, 2020
nxrB	nxrB169F	TACATGTGGTGGAACA	469	55	Nitrite reductase synthesis	Jiang *et al.*, 2020
	nxrB638R	CGGTTCTGGTCRATCA				
nirS	nirS2f	TACCACCCSGARCCGC GCGT	164	53	Denitrifica-tion NO_2^- to NO	Angnes *et al.*, 2013
	nirS3r	GCCGCCGTCRTGVAGG AA				
nosZ	nosZf	AGAACGACCAGCTGA TCGACA	380	53	Denitrifica-tion N_2O to N_2	Angnes *et al.*, 2013
	nosZr	TCCATGGTGACGCCGT GGTTG				

Supplementary Table S2 - Thermal conditions of the PCR reactions. Initial activation of polymerase for all reaction was at 95 °C during 5 minutes. There were 40 cycles in each case followed by 5 minute final extension at 72 °C

Gene	Cycle	Reference
ureC	95°C - 5 s, 50°C - 10 s, 72°C - 30 s	Oshiki *et al.*, 2018
amoA clade A	95°C - 30 s, 50°C - 30 s, 72°C - 30 s	Jiang *et al.*, 2020
amoA cladeB	95°C - 30 s, 50°C - 30 s, 72°C - 30 s	Jiang *et al.*, 2020
amoA	95°C - 15 s, 60°C - 60 s	Khangembam, Sharma and Chakrabarti, 2017
nirS	95°C - 15 s, 53°C - 15 s, 72°C - 60 s	Angnes *et al.*, 2013
nosZ	95°C - 15 s, 53°C - 15 s, 72°C - 60 s	Angnes *et al.*, 2013
nxrB	95°C - 30 s, 55°C - 45 s, 72°C - 45 s	Jiang *et al.*, 2020

Angnes, G. *et al.* (2013) 'Correlating denitrifying catabolic genes with N2O and N2 emissions from swine slurry composting', *Bioresource Technology*, 140, pp. 368–375. doi: https://doi.org/10.1016/j.biortech.2013.04.112.

Jiang, R. *et al.* (2020) 'Use of Newly Designed Primers for Quantification of Complete Ammonia-Oxidizing (Comammox) Bacterial Clades and Strict Nitrite Oxidizers in the Genus Nitrospira.', *Applied and environmental microbiology*, 86(20). doi: 10.1128/AEM.01775-20.

Khangembam, C. D., Sharma, J. G. and Chakrabarti, R. (2017) 'Diversity and Abundance of Ammonia-Oxidizing Bacteria and Archaea in a Freshwater Recirculating Aquaculture System', *HAYATI Journal of Biosciences*, 24(4), pp. 215–220. doi: https://doi.org/10.1016/j.hjb.2017.11.003.

Oshiki, M. *et al.* (2018) 'Ureolytic Prokaryotes in Soil: Community Abundance and Diversity.', *Microbes and environments*, 33(2), pp. 230–233. doi: 10.1264/jsme2.ME17188.

Supplementary Material

Supplementary Table S3 - OTUs identified as differentially abundant in moss banks impacted by varying intensities of penguin colonization. A general linear model with a negative binomial distribution was applied to identify significant relationships between OTUs and the intensity of penguin impact. P-values were corrected for multiple comparisons using the false discovery rate (q-value) method.

**OTU number**	**logFC**	**logCPM**	**LR**	**p-value**	**q-value**	**Phylum**	**Lowest taxonomic level**
OTU_2479	-3.75	6.75	6.67	9.8E-03	3.6E-02	Acidobacteriota	Acidobacteriae
OTU_825	-3.08	5.83	7.07	7.9E-03	3.0E-02	Acidobacteriota	Acidobacteriae
OTU_23	-2.53	12.71	10.39	1.3E-03	8.2E-03	Acidobacteriota	Acidobacteriales
OTU_4112	-4.52	10.12	7.15	7.5E-03	3.0E-02	Acidobacteriota	Subgroup_1
OTU_4213	-3.5	10.76	20.6	5.6E-06	1.9E-04	Acidobacteriota	Subgroup_1
OTU_4262	-2.98	5.91	6.34	1.2E-02	4.1E-02	Acidobacteriota	Subgroup_1
OTU_465	-3.07	7.87	16.68	4.4E-05	8.4E-04	Acidobacteriota	Subgroup_1
OTU_4853	-1.85	8.19	9.52	2.0E-03	1.1E-02	Acidobacteriota	Subgroup_1
OTU_83	-4.58	10.18	37.45	9.4E-10	4.3E-07	Acidobacteriota	Subgroup_1
OTU_11	-2.66	12.55	14.46	1.4E-04	1.9E-03	Acidobacteriota	Subgroup_1
OTU_129	-2.65	11.5	11.01	9.0E-04	6.5E-03	Acidobacteriota	Granulicella
OTU_3136	-3.81	7.97	25	5.7E-07	4.0E-05	Acidobacteriota	Granulicella
OTU_13	-3.74	13.17	22.78	1.8E-06	9.0E-05	Acidobacteriota	Granulicella
OTU_1431	-2.33	10.41	6.05	1.4E-02	4.7E-02	Acidobacteriota	Granulicella
OTU_38	-3.12	12.82	24.82	6.3E-07	4.3E-05	Acidobacteriota	Granulicella
OTU_54	-3.45	11.81	26	3.4E-07	3.1E-05	Acidobacteriota	Granulicella
OTU_2361	-2.95	10.1	19.34	1.1E-05	3.2E-04	Acidobacteriota	Occallatibacter
OTU_545	-3.16	8.35	17.1	3.5E-05	7.1E-04	Acidobacteriota	Occallatibacter
OTU_1599	-3.53	9	22.21	2.4E-06	1.1E-04	Acidobacteriota	Bryobacter
OTU_1689	-2.35	9.06	11.26	7.9E-04	5.9E-03	Acidobacteriota	Bryobacter
OTU_19	-2.17	12.77	6.94	8.4E-03	3.2E-02	Acidobacteriota	Bryobacter
OTU_2203	-3.77	5.9	10.09	1.5E-03	9.1E-03	Acidobacteriota	Bryobacter
OTU_248	-3.8	8.91	31.69	1.8E-08	3.9E-06	Acidobacteriota	Bryobacter
OTU_32	-2.73	11.7	19.39	1.1E-05	3.1E-04	Acidobacteriota	Bryobacter
OTU_3387	-2.89	8.34	13.91	1.9E-04	2.4E-03	Acidobacteriota	Bryobacter
OTU_4303	-2.23	8.83	7.21	7.2E-03	2.9E-02	Acidobacteriota	Bryobacter

OTU_4610	-3.26	5.65	12.07	5.1E-04	4.4E-03	Acidobacteriota	Bryobacter
OTU_5340	-4.09	5.93	8.99	2.7E-03	1.4E-02	Acidobacteriota	Bryobacter
OTU_6	-3.11	12.47	13.14	2.9E-04	3.1E-03	Acidobacteriota	Bryobacter
OTU_79	-3.28	10.74	13.1	3.0E-04	3.1E-03	Acidobacteriota	Bryobacter
OTU_837	-1.49	11.38	7.39	6.6E-03	2.7E-02	Acidobacteriota	Bryobacter
OTU_108	-1.85	10.17	6.09	1.4E-02	4.6E-02	Acidobacteriota	Candidatus_Solibacter
OTU_137	-2.03	9.81	10.47	1.2E-03	8.0E-03	Acidobacteriota	Candidatus_Solibacter
OTU_2860	-2.24	9.73	6.66	9.9E-03	3.6E-02	Acidobacteriota	Candidatus_Solibacter
OTU_3231	-4.71	9.97	12.29	4.6E-04	4.1E-03	Acidobacteriota	Candidatus_Solibacter
OTU_33	-2.22	11.4	6.21	1.3E-02	4.4E-02	Acidobacteriota	Candidatus_Solibacter
OTU_3888	-4.63	5.62	11.26	7.9E-04	5.9E-03	Acidobacteriota	Candidatus_Solibacter
OTU_4166	-3.18	9.43	8.58	3.4E-03	1.7E-02	Acidobacteriota	Candidatus_Solibacter
OTU_46	-2.79	12.35	22.6	2.0E-06	9.4E-05	Acidobacteriota	Candidatus_Solibacter
OTU_51	-2.94	10.47	9.64	1.9E-03	1.1E-02	Acidobacteriota	Candidatus_Solibacter
OTU_551	-1.81	8.01	7.25	7.1E-03	2.8E-02	Acidobacteriota	Candidatus_Solibacter
OTU_1197	-2.42	8.94	12.17	4.8E-04	4.3E-03	Acidobacteriota	Subgroup_2
OTU_14	-2.26	12.76	9.11	2.5E-03	1.3E-02	Acidobacteriota	Subgroup_2
OTU_22	-4.08	11.43	18.72	1.5E-05	3.8E-04	Acidobacteriota	Subgroup_2
OTU_3122	-3.36	11.8	18.33	1.9E-05	4.4E-04	Acidobacteriota	Subgroup_2
OTU_3688	-3.71	6.98	13.69	2.2E-04	2.6E-03	Acidobacteriota	Subgroup_2
OTU_3853	-2.26	8.73	9.52	2.0E-03	1.1E-02	Acidobacteriota	Subgroup_2
OTU_4135	-2.88	6.96	15.48	8.3E-05	1.3E-03	Acidobacteriota	Subgroup_2
OTU_4313	-5.09	11.3	16.19	5.7E-05	1.0E-03	Acidobacteriota	Subgroup_2
OTU_4622	-2.67	8.08	14.85	1.2E-04	1.6E-03	Acidobacteriota	Subgroup_2
OTU_481	-2.81	6.96	14.05	1.8E-04	2.2E-03	Acidobacteriota	Subgroup_2
OTU_703	-2.12	6.5	6.28	1.2E-02	4.2E-02	Acidobacteriota	Subgroup_2
OTU_87	-2.96	11.35	27.79	1.3E-07	1.5E-05	Acidobacteriota	Subgroup_2
OTU_1416	-4.35	5.59	8.04	4.6E-03	2.0E-02	Actinobacteriota	Acidimicrobiia
OTU_1634	4.6	5.6	10.46	1.2E-03	8.0E-03	Actinobacteriota	Acidimicrobiia
OTU_100	-2.15	11.14	10.17	1.4E-03	8.8E-03	Actinobacteriota	Acidimicrobiia
OTU_21	-3.75	11.84	32.42	1.2E-08	3.1E-06	Actinobacteriota	Acidimicrobiia
OTU_49	-3.11	10.69	11.82	5.9E-04	4.8E-03	Actinobacteriota	IMCC26256

OTU_762	-4.08	6.89	13.01	3.1E-04	3.2E-03	Actinobacteriota	IMCC26256
OTU_1420	6.6	5.95	11.34	7.6E-04	5.7E-03	Actinobacteriota	Actinobacteria
OTU_1947	3.32	5.36	6.59	1.0E-02	3.7E-02	Actinobacteriota	Actinobacteria
OTU_3197	4.81	5.84	8.81	3.0E-03	1.5E-02	Actinobacteriota	Actinobacteria
OTU_3764	6.67	6.03	14.01	1.8E-04	2.3E-03	Actinobacteriota	Actinobacteria
OTU_1834	-3.2	5.35	6.12	1.3E-02	4.5E-02	Actinobacteriota	Bifidobacterium_pseudolongum
OTU_139	-3.11	10.68	14.81	1.2E-04	1.6E-03	Actinobacteriota	Acidothermus
OTU_2553	-2.41	11.13	12.54	4.0E-04	3.8E-03	Actinobacteriota	Acidothermus
OTU_3154	3.07	9.71	8.55	3.5E-03	1.7E-02	Actinobacteriota	Acidothermus
OTU_3529	-3.02	6.63	6.13	1.3E-02	4.5E-02	Actinobacteriota	Acidothermus
OTU_926	6.58	6.69	10.47	1.2E-03	8.0E-03	Actinobacteriota	Micrococcales
OTU_1377	9.22	8.98	12.69	3.7E-04	3.6E-03	Actinobacteriota	Arthrobacter
OTU_2038	6.42	5.75	9.56	2.0E-03	1.1E-02	Actinobacteriota	Arthrobacter
OTU_2659	9.49	8.34	16.31	5.4E-05	9.8E-04	Actinobacteriota	Arthrobacter
OTU_4536	8.6	9.63	22.02	2.7E-06	1.2E-04	Actinobacteriota	Arthrobacter
OTU_2	8.64	16.06	19.47	1.0E-05	3.1E-04	Actinobacteriota	Arthrobacter
OTU_2143	6.92	13.45	18.83	1.4E-05	3.7E-04	Actinobacteriota	Arthrobacter
OTU_1418	5.66	5.5	9.8	1.7E-03	1.0E-02	Actinobacteriota	Gaiellales
OTU_1128	4.33	6.01	6.87	8.7E-03	3.3E-02	Actinobacteriota	Gaiellales
OTU_2449	-3.43	6.42	17.15	3.4E-05	7.0E-04	Actinobacteriota	Gaiellales
OTU_5932	4.14	4.88	6.73	9.5E-03	3.5E-02	Actinobacteriota	Conexibacter
OTU_358	7.66	8.09	12.21	4.7E-04	4.2E-03	Bacteroidota	Bacteroidales
OTU_5588	4.2	4.9	8.57	3.4E-03	1.7E-02	Bacteroidota	Bacteroidales
OTU_2871	3.38	4.75	6.41	1.1E-02	4.0E-02	Bacteroidota	Bacteroidetes
OTU_25	9.03	15.17	15.13	1.0E-04	1.5E-03	Bacteroidota	Proteiniphilum
OTU_2900	4.97	5.11	8.95	2.8E-03	1.4E-02	Bacteroidota	Proteiniphilum
OTU_2997	5.79	6.42	8.9	2.9E-03	1.5E-02	Bacteroidota	Proteiniphilum
OTU_3344	6.62	5.94	13.17	2.8E-04	3.1E-03	Bacteroidota	Proteiniphilum
OTU_4954	6.97	6.97	11.95	5.5E-04	4.6E-03	Bacteroidota	Proteiniphilum
OTU_325	5.78	8.11	8.4	3.8E-03	1.8E-02	Bacteroidota	Chitinophagales
OTU_3360	-5.11	5.02	8.04	4.6E-03	2.0E-02	Bacteroidota	Chitinophagales

OTU_44	-5.3	11.26	25.57	4.3E-07	3.5E-05	Bacteroidota	Chitinophagales
OTU_1302	3.89	5.05	6.13	1.3E-02	4.5E-02	Bacteroidota	Chitinophagales
OTU_741	-3.25	6.6	17.21	3.3E-05	6.8E-04	Bacteroidota	Chitinophagales
OTU_865	5.07	7.39	23	1.6E-06	8.7E-05	Bacteroidota	Chitinophagales
OTU_1066	6.38	5.75	8.66	3.2E-03	1.6E-02	Bacteroidota	Chitinophagales
OTU_1322	6.92	6.14	13.55	2.3E-04	2.7E-03	Bacteroidota	Chitinophagales
OTU_1294	3.93	5.36	7.92	4.9E-03	2.1E-02	Bacteroidota	Chitinophagaceae
OTU_1662	-2.59	5.62	7.89	5.0E-03	2.2E-02	Bacteroidota	Chitinophagaceae
OTU_3222	-3	6.5	8.61	3.3E-03	1.6E-02	Bacteroidota	Chitinophagaceae
OTU_374	4.44	8.26	11.48	7.0E-04	5.4E-03	Bacteroidota	Chitinophagaceae
OTU_658	5.09	6.74	13.16	2.9E-04	3.1E-03	Bacteroidota	Chitinophagaceae
OTU_661	4.77	5.98	8.35	3.9E-03	1.8E-02	Bacteroidota	Chitinophagaceae
OTU_687	-5.15	6.13	11.94	5.5E-04	4.6E-03	Bacteroidota	Chitinophagaceae
OTU_935	6.53	6.06	11.3	7.8E-04	5.8E-03	Bacteroidota	Chitinophagaceae
OTU_3177	4.82	5.11	7.14	7.5E-03	3.0E-02	Bacteroidota	Chitinophagaceae
OTU_462	8.57	11.6	11.49	7.0E-04	5.4E-03	Bacteroidota	Chitinophagaceae
OTU_4923	4.64	5.09	8.55	3.5E-03	1.7E-02	Bacteroidota	Chitinophagaceae
OTU_5182	3.66	4.79	6.65	9.9E-03	3.6E-02	Bacteroidota	Chitinophagaceae
OTU_1335	5.27	7.11	7.8	5.2E-03	2.2E-02	Bacteroidota	Chitinophagaceae
OTU_2137	3.64	7.24	6.52	1.1E-02	3.8E-02	Bacteroidota	Chitinophagaceae
OTU_24	7.75	13.83	21.55	3.4E-06	1.3E-04	Bacteroidota	Chitinophagaceae
OTU_989	5.61	6.18	9.54	2.0E-03	1.1E-02	Bacteroidota	Chitinophagaceae
OTU_1669	-2.26	8.17	7.25	7.1E-03	2.8E-02	Bacteroidota	Puia
OTU_5568	-3.17	9.29	13.23	2.8E-04	3.0E-03	Bacteroidota	Puia
OTU_2202	5.55	5.53	11.52	6.9E-04	5.3E-03	Bacteroidota	Flavobacteriales
OTU_61	9.3	12.76	16.68	4.4E-05	8.4E-04	Bacteroidota	Aequorivita
OTU_931	7.86	10.06	14.41	1.5E-04	1.9E-03	Bacteroidota	Aequorivita
OTU_2822	6.45	5.78	8.47	3.6E-03	1.7E-02	Bacteroidota	Aequorivita
OTU_1109	6.15	5.73	12.24	4.7E-04	4.2E-03	Bacteroidota	Flavobacterium
OTU_180	8.89	10.69	27.62	1.5E-07	1.5E-05	Bacteroidota	Flavobacterium
OTU_1810	4.25	4.92	7.14	7.5E-03	3.0E-02	Bacteroidota	Flavobacterium
OTU_1885	5.35	7.12	20.91	4.8E-06	1.7E-04	Bacteroidota	Flavobacterium

OTU_2065	3.67	7.23	13.17	2.9E-04	3.1E-03	Bacteroidota	Flavobacterium
OTU_2539	7.1	7.12	15.83	6.9E-05	1.2E-03	Bacteroidota	Flavobacterium
OTU_4607	5.46	6.83	21.73	3.1E-06	1.3E-04	Bacteroidota	Flavobacterium
OTU_469	5.38	7.54	12.56	3.9E-04	3.8E-03	Bacteroidota	Flavobacterium
OTU_755	7.78	7.81	31.27	2.2E-08	4.5E-06	Bacteroidota	Flavobacterium
OTU_763	6.88	6.88	9.07	2.6E-03	1.4E-02	Bacteroidota	Flavobacterium
OTU_815	4.69	5.82	11.29	7.8E-04	5.9E-03	Bacteroidota	Flavobacterium
OTU_830	4.18	6.11	13.11	2.9E-04	3.1E-03	Bacteroidota	Flavobacterium
OTU_886	6.1	6.28	9.56	2.0E-03	1.1E-02	Bacteroidota	Flavobacterium
OTU_892	7.28	6.39	17.32	3.2E-05	6.6E-04	Bacteroidota	Flavobacterium
OTU_195	8.02	10.76	29.95	4.4E-08	7.5E-06	Bacteroidota	Flavobacterium
OTU_445	8.7	8.59	25.53	4.4E-07	3.5E-05	Bacteroidota	Flavobacterium
OTU_28	8.22	12.56	28.26	1.1E-07	1.3E-05	Bacteroidota	Flavobacterium
OTU_1133	4.18	6.11	10.73	1.1E-03	7.1E-03	Bacteroidota	Flavobacterium
OTU_88	9.46	13.11	26.47	2.7E-07	2.5E-05	Bacteroidota	Gelidibacter
OTU_35	7.72	12.92	17.99	2.2E-05	4.9E-04	Bacteroidota	Chryseobacterium
OTU_369	4.34	8.45	7.27	7.0E-03	2.8E-02	Bacteroidota	Chryseobacterium
OTU_3931	7.58	10.16	22.65	1.9E-06	9.4E-05	Bacteroidota	Chryseobacterium
OTU_676	5.68	9.24	16.02	6.3E-05	1.1E-03	Bacteroidota	Chryseobacterium
OTU_190	5.57	11.89	18.22	2.0E-05	4.6E-04	Bacteroidota	Chryseobacterium
OTU_3501	5.17	5.51	8.89	2.9E-03	1.5E-02	Bacteroidota	Sphingobacteriaceae
OTU_455	6.29	8.43	17.79	2.5E-05	5.3E-04	Bacteroidota	Sphingobacteriaceae
OTU_177	-5.08	9.02	22.21	2.4E-06	1.1E-04	Bacteroidota	Mucilaginibacter
OTU_230	-2.38	8.97	7.99	4.7E-03	2.1E-02	Bacteroidota	Mucilaginibacter
OTU_237	-3.23	8.56	18.93	1.4E-05	3.7E-04	Bacteroidota	Mucilaginibacter
OTU_2872	-4.21	7.95	24.72	6.6E-07	4.4E-05	Bacteroidota	Mucilaginibacter
OTU_3189	-3.95	6.91	11.66	6.4E-04	5.1E-03	Bacteroidota	Mucilaginibacter
OTU_4055	-6.17	5.47	11.97	5.4E-04	4.5E-03	Bacteroidota	Mucilaginibacter
OTU_793	-5	7.19	15.28	9.3E-05	1.4E-03	Bacteroidota	Mucilaginibacter
OTU_2972	-2.67	7.84	6.28	1.2E-02	4.2E-02	Bacteroidota	Mucilaginibacter
OTU_370	-3.96	8.97	13.04	3.1E-04	3.2E-03	Bacteroidota	Mucilaginibacter

OTU_625	-4.15	8.28	15.33	9.0E-05	1.4E-03	Bacteroidota	Mucilaginibacter
OTU_904	-3.17	7.31	10.5	1.2E-03	7.9E-03	Bacteroidota	Mucilaginibacter
OTU_1568	5.7	5.44	11.84	5.8E-04	4.8E-03	Bacteroidota	Mucilaginibacter
OTU_1932	7.47	10.42	29.58	5.4E-08	8.7E-06	Bacteroidota	Pedobacter
OTU_215	9.48	11.25	17.3	3.2E-05	6.6E-04	Bacteroidota	Pedobacter
OTU_2184	6.2	8.99	19.65	9.3E-06	2.8E-04	Bacteroidota	Pedobacter
OTU_219	9.55	10.22	13.19	2.8E-04	3.1E-03	Bacteroidota	Pedobacter
OTU_233	8.2	9.97	23.24	1.4E-06	8.3E-05	Bacteroidota	Pedobacter
OTU_2550	5.94	9.71	20.02	7.7E-06	2.4E-04	Bacteroidota	Pedobacter
OTU_275	5.36	8.26	17.37	3.1E-05	6.5E-04	Bacteroidota	Pedobacter
OTU_341	7.09	8.61	11.69	6.3E-04	5.0E-03	Bacteroidota	Pedobacter
OTU_3687	3.58	7.22	6.88	8.7E-03	3.3E-02	Bacteroidota	Pedobacter
OTU_441	6.02	9.81	20.93	4.8E-06	1.7E-04	Bacteroidota	Pedobacter
OTU_585	7.43	7.88	14.93	1.1E-04	1.6E-03	Bacteroidota	Pedobacter
OTU_626	6.68	7.36	11.55	6.8E-04	5.3E-03	Bacteroidota	Pedobacter
OTU_724	4.67	7.89	12.16	4.9E-04	4.3E-03	Bacteroidota	Pedobacter
OTU_3063	4.42	7.34	10.19	1.4E-03	8.7E-03	Bacteroidota	Pedobacter
OTU_3590	9.19	9	22.35	2.3E-06	1.0E-04	Bacteroidota	Pedobacter
OTU_637	4.76	7.57	11.9	5.6E-04	4.7E-03	Bacteroidota	Pedobacter
OTU_130	6.05	11.28	28.14	1.1E-07	1.3E-05	Bacteroidota	Pedobacter
OTU_454	6.87	8.91	22.95	1.7E-06	8.7E-05	Bacteroidota	Pedobacter
OTU_578	6.52	8.55	13.65	2.2E-04	2.6E-03	Bacteroidota	Pedobacter
OTU_2500	-4.22	6.16	7.66	5.6E-03	2.4E-02	Chloroflexi	AD3;
OTU_68	-3.36	10.48	16.82	4.1E-05	7.9E-04	Chloroflexi	AD3;
OTU_89	-2.78	10.55	22.96	1.7E-06	8.7E-05	Chloroflexi	AD3;
OTU_744	5.85	6.19	8.82	3.0E-03	1.5E-02	Chloroflexi	A4b
OTU_932	4.4	5.6	6.1	1.4E-02	4.6E-02	Chloroflexi	Herpetosiphon
OTU_890	5.29	6.13	6.44	1.1E-02	4.0E-02	Chloroflexi	Herpetosiphon
OTU_1306	4.1	5.48	6.3	1.2E-02	4.2E-02	Chloroflexi	Roseiflexaceae
OTU_1323	4.45	5.77	7.06	7.9E-03	3.1E-02	Chloroflexi	Roseiflexaceae
OTU_1848	4.38	5	6.18	1.3E-02	4.4E-02	Chloroflexi	Roseiflexaceae
OTU_1063	4.08	5.62	6.58	1.0E-02	3.7E-02	Chloroflexi	JG30-KF-CM45

OTU_2071	4.62	5	8.84	2.9E-03	1.5E-02	Chloroflexi	JG30-KF-CM45
OTU_2342	6.04	5.63	15.89	6.7E-05	1.1E-03	Chloroflexi	JG30-KF-CM45
OTU_117	-4.28	9.97	16.26	5.5E-05	1.0E-03	Chloroflexi	Ktedonobacteraceae
OTU_450	6.71	8.25	11.99	5.3E-04	4.5E-03	Chloroflexi	Ktedonobacteraceae
OTU_1194	6.03	5.79	7.86	5.1E-03	2.2E-02	Chloroflexi	Ktedonobacteraceae
OTU_612	5.09	8.84	7.64	5.7E-03	2.4E-02	Chloroflexi	Ktedonobacteraceae
OTU_2640	6.51	5.95	11.8	5.9E-04	4.8E-03	Cyanobacteria	Tychonema_CCAP_1459-11B
OTU_3	7.03	15.21	12.35	4.4E-04	4.0E-03	Cyanobacteria	Tychonema_CCAP_1459-11B
OTU_530	3.97	7.28	6.44	1.1E-02	4.0E-02	Cyanobacteria	Tychonema_CCAP_1459-11B
OTU_91	5.64	10.81	10.93	9.4E-04	6.7E-03	Cyanobacteria	Leptolyngbyaceae
OTU_29	7.79	12.8	20.65	5.5E-06	1.9E-04	Firmicutes	Sporosarcina
OTU_4894	3.82	5.23	6.14	1.3E-02	4.5E-02	Firmicutes	Sporosarcina
OTU_524	6.03	8.93	18.5	1.7E-05	4.1E-04	Firmicutes	Sporosarcina
OTU_2719	5.6	7.89	11.85	5.8E-04	4.7E-03	Firmicutes	Sporosarcina
OTU_759	2.97	7.01	11.72	6.2E-04	5.0E-03	Firmicutes	Sporosarcina
OTU_4023	5.31	9.89	8.73	3.1E-03	1.6E-02	Firmicutes	Ruminococcaceae
OTU_338	-6.46	8.29	6.33	1.2E-02	4.2E-02	Firmicutes	Ruminococcaceae
OTU_15	7.19	13.42	9.66	1.9E-03	1.1E-02	Firmicutes	Gottschalkia
OTU_3003	3.62	4.79	10.23	1.4E-03	8.6E-03	Firmicutes	Gottschalkia
OTU_3346	5.31	5.24	9.39	2.2E-03	1.2E-02	Firmicutes	Gottschalkia
OTU_3884	6.18	5.68	10.08	1.5E-03	9.1E-03	Firmicutes	Gottschalkia
OTU_419	6.6	8.32	11.2	8.2E-04	6.0E-03	Firmicutes	Gottschalkia
OTU_5081	5.86	7.1	10.75	1.0E-03	7.1E-03	Firmicutes	Gottschalkia
OTU_5761	5.8	5.48	12.62	3.8E-04	3.7E-03	Firmicutes	Gottschalkia
OTU_77	6.52	12.26	18.03	2.2E-05	4.9E-04	Firmicutes	Gottschalkia
OTU_909	4.57	7.86	11.59	6.6E-04	5.2E-03	Firmicutes	Gottschalkia
OTU_2806	8.2	11.71	10.28	1.3E-03	8.5E-03	Firmicutes	Tissierella
OTU_4329	7.64	9.44	14.99	1.1E-04	1.5E-03	Firmicutes	Tissierella
OTU_4920	4.29	4.91	7.3	6.9E-03	2.8E-02	Firmicutes	Tissierella
OTU_5046	6.16	5.67	11.5	7.0E-04	5.4E-03	Firmicutes	Tissierella
OTU_1382	6.23	9.19	10.72	1.1E-03	7.1E-03	Firmicutes	Tissierella

OTU_145	7.18	11.27	9.37	2.2E-03	1.2E-02	Firmicutes	Tissierella
OTU_1887	6.53	5.89	15.69	7.4E-05	1.2E-03	Firmicutes	Tissierella
OTU_2273	7.83	9.68	10.71	1.1E-03	7.2E-03	Firmicutes	Tissierella
OTU_4104	4.62	5.02	6.94	8.4E-03	3.2E-02	Firmicutes	Tissierella
OTU_4160	7.07	6.27	12.8	3.5E-04	3.4E-03	Firmicutes	Tissierella
OTU_43	7.27	12.46	17.69	2.6E-05	5.6E-04	Firmicutes	Tissierella
OTU_862	8.26	7.25	13.95	1.9E-04	2.3E-03	Firmicutes	Tissierella
OTU_99	7.64	12.25	14.79	1.2E-04	1.7E-03	Firmicutes	Tissierella
OTU_1279	4.01	5.31	7.87	5.0E-03	2.2E-02	Gemmatimonadota	Gemmatimonadaceae
OTU_149	-2.48	9.74	6.57	1.0E-02	3.8E-02	Gemmatimonadota	Gemmatimonadaceae
OTU_36	-2.01	11.11	6.89	8.7E-03	3.3E-02	Gemmatimonadota	Gemmatimonadaceae
OTU_262	6.88	9.71	8.17	4.3E-03	1.9E-02	Gemmatimonadota	Gemmatimonas
OTU_395	-2.98	7.02	7.31	6.8E-03	2.7E-02	Gemmatimonadota	Gemmatimonas
OTU_852	-3.57	5.64	8.19	4.2E-03	1.9E-02	Gemmatimonadota	Gemmatimonas
OTU_146	-2.66	9.35	6.27	1.2E-02	4.2E-02	Gemmatimonadota	Gemmatimonas
OTU_183	3.49	10.17	12.07	5.1E-04	4.4E-03	Gemmatimonadota	Gemmatimonas
OTU_297	-3.03	7.97	9.16	2.5E-03	1.3E-02	Gemmatimonadota	Gemmatimonas
OTU_422	4.63	7.86	7.63	5.7E-03	2.4E-02	Gemmatimonadota	Gemmatimonas
OTU_470	-2.81	7.05	10.38	1.3E-03	8.2E-03	Gemmatimonadota	Gemmatimonas
OTU_1105	-2.98	7.02	16.86	4.0E-05	7.9E-04	Myxococcota	Pajaroellobacter
OTU_128	-3.19	10.24	15.73	7.3E-05	1.2E-03	Myxococcota	Pajaroellobacter
OTU_1498	-4.52	5.31	10.85	9.9E-04	6.8E-03	Myxococcota	Pajaroellobacter
OTU_217	-3.46	8.93	15.11	1.0E-04	1.5E-03	Myxococcota	Pajaroellobacter
OTU_240	-4.13	9.1	23.24	1.4E-06	8.3E-05	Myxococcota	Pajaroellobacter
OTU_313	4.03	8.02	9.83	1.7E-03	1.0E-02	Myxococcota	Pajaroellobacter
OTU_3246	-3.86	5.37	8.93	2.8E-03	1.4E-02	Myxococcota	Pajaroellobacter
OTU_348	-3.4	8.19	15.65	7.6E-05	1.2E-03	Myxococcota	Pajaroellobacter
OTU_416	-4.76	7.2	13.75	2.1E-04	2.5E-03	Myxococcota	Pajaroellobacter
OTU_498	-4.84	6.86	21.39	3.7E-06	1.4E-04	Myxococcota	Pajaroellobacter
OTU_702	-5.08	6.06	11.23	8.0E-04	6.0E-03	Myxococcota	Pajaroellobacter
OTU_93	-3.41	10.16	15.78	7.1E-05	1.2E-03	Myxococcota	Pajaroellobacter
OTU_930	-5.61	6.07	16.94	3.8E-05	7.6E-04	Myxococcota	Pajaroellobacter

OTU_1000	-5.11	6.41	11.07	8.8E-04	6.3E-03	Planctomycetota	WD2101_soil_group
OTU_1057	-4.03	5.35	6.25	1.2E-02	4.3E-02	Planctomycetota	WD2101_soil_group
OTU_1235	-4.63	5.16	8.63	3.3E-03	1.6E-02	Planctomycetota	WD2101_soil_group
OTU_1372	-2.83	5.39	6.37	1.2E-02	4.1E-02	Planctomycetota	WD2101_soil_group
OTU_1421	-4.4	5.2	6.78	9.2E-03	3.4E-02	Planctomycetota	WD2101_soil_group
OTU_1477	-4.15	5.36	8.9	2.9E-03	1.5E-02	Planctomycetota	WD2101_soil_group
OTU_1690	-3.45	4.99	6.55	1.0E-02	3.8E-02	Planctomycetota	WD2101_soil_group
OTU_296	-6.61	8.47	40.89	1.6E-10	1.0E-07	Planctomycetota	WD2101_soil_group
OTU_3133	-4.72	6.77	8.74	3.1E-03	1.6E-02	Planctomycetota	WD2101_soil_group
OTU_4256	-6.06	7.7	25.81	3.8E-07	3.3E-05	Planctomycetota	WD2101_soil_group
OTU_675	-3.19	7.19	13.49	2.4E-04	2.8E-03	Planctomycetota	WD2101_soil_group
OTU_752	-3.5	7.37	15.78	7.1E-05	1.2E-03	Planctomycetota	WD2101_soil_group
OTU_81	-6.36	10.73	25.23	5.1E-07	3.6E-05	Planctomycetota	WD2101_soil_group
OTU_1554	-4.04	5.01	8.66	3.2E-03	1.6E-02	Planctomycetota	Gemmataceae
OTU_3174	-2.91	7.25	9.35	2.2E-03	1.2E-02	Planctomycetota	Gemmataceae
OTU_1910	-3.38	6.21	13.2	2.8E-04	3.1E-03	Planctomycetota	Candidatus_Nostocoida
OTU_2145	3.98	4.93	7.08	7.8E-03	3.0E-02	Planctomycetota	Paludisphaera
OTU_1793	-4.94	5.46	12.27	4.6E-04	4.2E-03	Planctomycetota	Tundrisphaera
OTU_1854	-4.33	5.02	6.77	9.3E-03	3.4E-02	Planctomycetota	Pirellulaceae
OTU_2370	5.21	5.17	7.75	5.4E-03	2.3E-02	Planctomycetota	Pirellulaceae
OTU_1842	-4.06	4.84	5.93	1.5E-02	5.0E-02	Alphaproteobacteria	Alphaproteobacteria
OTU_402	-4.35	7.1	6.08	1.4E-02	4.6E-02	Alphaproteobacteria	Alphaproteobacteria
OTU_160	-4.15	9.2	7.87	5.0E-03	2.2E-02	Alphaproteobacteria	Acetobacteraceae
OTU_2127	-3.02	8.9	7.38	6.6E-03	2.7E-02	Alphaproteobacteria	Acetobacteraceae
OTU_3958	-3.49	10.63	34.69	3.9E-09	1.3E-06	Alphaproteobacteria	Acetobacteraceae
OTU_4012	-3.22	5.45	7.48	6.3E-03	2.6E-02	Alphaproteobacteria	Acetobacteraceae
OTU_4715	-4.06	7.2	19.29	1.1E-05	3.2E-04	Alphaproteobacteria	Acetobacteraceae
OTU_998	-2.47	10.02	10.1	1.5E-03	9.1E-03	Alphaproteobacteria	Acetobacteraceae
OTU_142	-2.62	10.95	10.97	9.2E-04	6.6E-03	Alphaproteobacteria	Acetobacteraceae
OTU_1547	4.3	5.45	12.86	3.4E-04	3.4E-03	Alphaproteobacteria	Acidiphilium
OTU_3731	-4.36	5.49	19.43	1.0E-05	3.1E-04	Alphaproteobacteria	Caulobacteraceae

OTU_3694	-4.47	6.51	8	4.7E-03	2.1E-02	Alphaproteobacteria	Caulobacteraceae
OTU_1409	-3.95	5.17	10.95	9.4E-04	6.6E-03	Alphaproteobacteria	Caulobacteraceae
OTU_228	-3.47	8.57	24.28	8.3E-07	5.4E-05	Alphaproteobacteria	Caulobacteraceae
OTU_110	-2.63	8.91	10.86	9.8E-04	6.8E-03	Alphaproteobacteria	Elsterales
OTU_418	-4.1	7.04	33.31	7.8E-09	2.3E-06	Alphaproteobacteria	Elsterales
OTU_443	-3.11	7.03	8.7	3.2E-03	1.6E-02	Alphaproteobacteria	Elsterales
OTU_713	-3.96	5.75	15.35	8.9E-05	1.4E-03	Alphaproteobacteria	Elsterales
OTU_2020	-5.94	8.56	25.42	4.6E-07	3.5E-05	Alphaproteobacteria	Micropepsaceae
OTU_5936	-4	5.49	8.02	4.6E-03	2.1E-02	Alphaproteobacteria	Micropepsaceae
OTU_186	-3.02	9.71	18.93	1.4E-05	3.7E-04	Alphaproteobacteria	Micropepsaceae
OTU_2866	-4.04	6.12	12.48	4.1E-04	3.8E-03	Alphaproteobacteria	Micropepsaceae
OTU_5478	-4.78	5.21	8.69	3.2E-03	1.6E-02	Alphaproteobacteria	Micropepsaceae
OTU_121	-2.19	9.42	11.22	8.1E-04	6.0E-03	Alphaproteobacteria	Micropepsaceae
OTU_1390	-3.28	9.02	28.54	9.2E-08	1.3E-05	Alphaproteobacteria	Micropepsaceae
OTU_345	-3.09	9.51	9.09	2.6E-03	1.4E-02	Alphaproteobacteria	Micropepsaceae
OTU_4076	-2.81	9.11	21.95	2.8E-06	1.2E-04	Alphaproteobacteria	Micropepsaceae
OTU_4396	-3.26	9.18	21.5	3.5E-06	1.4E-04	Alphaproteobacteria	Micropepsaceae
OTU_473	-4.3	8.15	15.58	7.9E-05	1.3E-03	Alphaproteobacteria	Micropepsaceae
OTU_5222	-3.28	6.43	9.9	1.7E-03	9.9E-03	Alphaproteobacteria	Micropepsaceae
OTU_80	-2.95	11.57	35.6	2.4E-09	8.7E-07	Alphaproteobacteria	Micropepsaceae
OTU_3619	-2.3	9.31	12.54	4.0E-04	3.8E-03	Alphaproteobacteria	Roseiarcus
OTU_495	-1.63	8.61	6.09	1.4E-02	4.6E-02	Alphaproteobacteria	Roseiarcus
OTU_176	-3.04	9.44	13.52	2.4E-04	2.7E-03	Alphaproteobacteria	Rhizobiales_Incertae_Sedis
OTU_563	-1.93	6.55	6.43	1.1E-02	4.0E-02	Alphaproteobacteria	Rhizobiales_Incertae_Sedis
OTU_5368	-3.49	6.14	8.37	3.8E-03	1.8E-02	Alphaproteobacteria	Xanthobacteraceae
OTU_2382	-2.09	8.25	8.56	3.4E-03	1.7E-02	Alphaproteobacteria	Xanthobacteraceae
OTU_3611	-3.69	4.78	8.06	4.5E-03	2.0E-02	Alphaproteobacteria	Pseudolabrys
OTU_48	-3.16	12.53	29.06	7.0E-08	1.1E-05	Alphaproteobacteria	Pseudolabrys
OTU_164	5.34	9.77	11.04	8.9E-04	6.4E-03	Alphaproteobacteria	Rhodobacteraceae
OTU_1917	4.39	4.97	6.47	1.1E-02	3.9E-02	Alphaproteobacteria	Rhodobacteraceae
OTU_2135	3.59	4.78	6.53	1.1E-02	3.8E-02	Alphaproteobacteria	Rhodobacteraceae
OTU_601	4.33	8.36	9.21	2.4E-03	1.3E-02	Alphaproteobacteria	Rhodobacteraceae

OTU_696	5.86	8.49	12.2	4.8E-04	4.2E-03	Alphaproteobacteria	Rhodobacteraceae
OTU_2618	3.38	4.79	6.52	1.1E-02	3.8E-02	Betaproteobacteria	Burkholderiales
OTU_701	-3.25	7	9.32	2.3E-03	1.2E-02	Betaproteobacteria	Burkholderiales
OTU_901	2.78	7.07	7.93	4.9E-03	2.1E-02	Betaproteobacteria	Burkholderiales
OTU_1577	4.7	6.97	13.63	2.2E-04	2.6E-03	Betaproteobacteria	Comamonadaceae
OTU_1666	7.95	7.94	13.33	2.6E-04	2.9E-03	Betaproteobacteria	Comamonadaceae
OTU_2097	4.85	6.23	9.32	2.3E-03	1.2E-02	Betaproteobacteria	Comamonadaceae
OTU_2300	7.3	12.1	25.43	4.6E-07	3.5E-05	Betaproteobacteria	Comamonadaceae
OTU_2490	5.64	9.12	23.08	1.6E-06	8.7E-05	Betaproteobacteria	Comamonadaceae
OTU_2985	7.56	6.67	20.54	5.8E-06	1.9E-04	Betaproteobacteria	Comamonadaceae
OTU_3021	6.81	6.99	10.91	9.6E-04	6.7E-03	Betaproteobacteria	Comamonadaceae
OTU_3077	3.46	7.45	8.01	4.6E-03	2.1E-02	Betaproteobacteria	Comamonadaceae
OTU_3111	7.7	6.79	21.39	3.8E-06	1.4E-04	Betaproteobacteria	Comamonadaceae
OTU_3416	3.86	4.83	9.29	2.3E-03	1.2E-02	Betaproteobacteria	Comamonadaceae
OTU_3868	4.08	6.11	13.66	2.2E-04	2.6E-03	Betaproteobacteria	Comamonadaceae
OTU_4125	6.12	8.61	25.43	4.6E-07	3.5E-05	Betaproteobacteria	Comamonadaceae
OTU_4582	4.35	4.93	7.15	7.5E-03	3.0E-02	Betaproteobacteria	Comamonadaceae
OTU_476	3.98	7.92	15.64	7.7E-05	1.2E-03	Betaproteobacteria	Comamonadaceae
OTU_5188	4.67	6.28	12.9	3.3E-04	3.3E-03	Betaproteobacteria	Comamonadaceae
OTU_5195	6.04	5.61	13.16	2.9E-04	3.1E-03	Betaproteobacteria	Comamonadaceae
OTU_589	5.54	8.07	15.31	9.1E-05	1.4E-03	Betaproteobacteria	Comamonadaceae
OTU_643	3.98	7.79	12.53	4.0E-04	3.8E-03	Betaproteobacteria	Comamonadaceae
OTU_2335	4.04	8.71	9.58	2.0E-03	1.1E-02	Betaproteobacteria	Polaromonas
OTU_2916	3.71	4.85	6.81	9.1E-03	3.4E-02	Betaproteobacteria	Polaromonas
OTU_4059	4.31	4.97	6.85	8.9E-03	3.3E-02	Betaproteobacteria	Polaromonas
OTU_4265	4.23	4.94	12.57	3.9E-04	3.7E-03	Betaproteobacteria	Polaromonas
OTU_945	5.98	12.29	22.85	1.7E-06	9.0E-05	Betaproteobacteria	Polaromonas
OTU_489	5.73	9.42	22.8	1.8E-06	9.0E-05	Betaproteobacteria	Polaromonas
OTU_69	4.78	12.81	21	4.6E-06	1.6E-04	Betaproteobacteria	Polaromonas
**OTU_1**	8.62	17.60	16.15	5.8E-05	1.0E-03	Betaproteobacteria	Rhodoferax
OTU_1555	7.87	8.43	12.42	4.2E-04	3.9E-03	Betaproteobacteria	Rhodoferax

OTU_3385	6.81	6.09	13.87	2.0E-04	2.4E-03	Betaproteobacteria	Rhodoferax
OTU_4069	5.39	5.26	8.07	4.5E-03	2.0E-02	Betaproteobacteria	Rhodoferax
OTU_4529	6.76	6.07	15.32	9.1E-05	1.4E-03	Betaproteobacteria	Rhodoferax
OTU_488	4.74	10.46	17.84	2.4E-05	5.2E-04	Betaproteobacteria	Rhodoferax
OTU_243	5.31	11.68	20.91	4.8E-06	1.7E-04	Betaproteobacteria	Rhodoferax
OTU_1590	6.74	6.03	16.38	5.2E-05	9.6E-04	Betaproteobacteria	Rhodoferax
OTU_2144	6.04	5.6	9.45	2.1E-03	1.2E-02	Betaproteobacteria	Rhodoferax
OTU_2852	4.02	4.86	8.5	3.6E-03	1.7E-02	Betaproteobacteria	Rhodoferax
OTU_3542	8.46	7.44	11.37	7.5E-04	5.6E-03	Betaproteobacteria	Rhodoferax
OTU_4006	8.53	11.59	15.11	1.0E-04	1.5E-03	Betaproteobacteria	Rhodoferax
OTU_4845	7.28	7.33	15.37	8.8E-05	1.4E-03	Betaproteobacteria	Rhodoferax
OTU_4922	5.31	5.25	10.21	1.4E-03	8.7E-03	Betaproteobacteria	Rhodoferax
OTU_5484	7.64	11.25	10.63	1.1E-03	7.5E-03	Betaproteobacteria	Rhodoferax
OTU_3915	3.48	4.77	6.27	1.2E-02	4.2E-02	Betaproteobacteria	Simplicispira
OTU_20	6.84	14.01	21.94	2.8E-06	1.2E-04	Betaproteobacteria	Simplicispira
OTU_239	7.25	12.4	22.65	1.9E-06	9.4E-05	Betaproteobacteria	Simplicispira
OTU_1156	5.25	6.27	11.86	5.7E-04	4.7E-03	Betaproteobacteria	Oxalobacteraceae
OTU_266	6.38	9.56	8.92	2.8E-03	1.5E-02	Betaproteobacteria	Oxalobacteraceae
OTU_366	3.65	7.67	10.92	9.5E-04	6.7E-03	Betaproteobacteria	Oxalobacteraceae
OTU_376	3.86	8.43	15.94	6.5E-05	1.1E-03	Betaproteobacteria	Oxalobacteraceae
OTU_3768	5.36	6.61	13.06	3.0E-04	3.1E-03	Betaproteobacteria	Oxalobacteraceae
OTU_5837	-3.92	5.03	8.3	4.0E-03	1.8E-02	Betaproteobacteria	Oxalobacteraceae
OTU_4562	5.88	6.22	8.83	3.0E-03	1.5E-02	Gammaproteobacteria	Psychrobacter
OTU_5636	5.48	5.37	9.01	2.7E-03	1.4E-02	Gammaproteobacteria	Psychrobacter
OTU_76	6.77	12.24	9.02	2.7E-03	1.4E-02	Gammaproteobacteria	Psychrobacter
OTU_75	5.86	13.18	19.13	1.2E-05	3.4E-04	Gammaproteobacteria	Psychrobacter
OTU_1368	6.8	11.54	13.46	2.4E-04	2.8E-03	Gammaproteobacteria	Psychrobacter
OTU_143	-2.75	9.68	9	2.7E-03	1.4E-02	Gammaproteobacteria	WD260;
OTU_52	-2.68	11.85	16.83	4.1E-05	7.9E-04	Gammaproteobacteria	WD260;
OTU_845	-2.78	11.56	18.65	1.6E-05	3.9E-04	Gammaproteobacteria	WD260;
OTU_772	7.22	7.97	10.24	1.4E-03	8.6E-03	Gammaproteobacteria	Rhodanobacteraceae

OTU_3374	2.82	5.64	7.05	7.9E-03	3.1E-02	Gammaproteobacteria	Dokdonella
OTU_73	6.49	12.37	15.97	6.4E-05	1.1E-03	Gammaproteobacteria	Dokdonella
OTU_131	5.73	10.1	16.45	5.0E-05	9.3E-04	Gammaproteobacteria	Dokdonella
OTU_140	3.89	10.48	9.27	2.3E-03	1.3E-02	Gammaproteobacteria	Dokdonella
OTU_3336	3.6	4.77	7.27	7.0E-03	2.8E-02	Gammaproteobacteria	Dokdonella
OTU_4967	2.97	7.83	9.73	1.8E-03	1.0E-02	Gammaproteobacteria	Dokdonella
OTU_3948	4.44	4.97	8.84	2.9E-03	1.5E-02	Gammaproteobacteria	Xanthomonadaceae
OTU_429	6.09	7.75	11.69	6.3E-04	5.0E-03	Gammaproteobacteria	Xanthomonadaceae
OTU_552	4.26	7.24	7.47	6.3E-03	2.6E-02	Gammaproteobacteria	Xanthomonadaceae
OTU_900	-3.67	6.2	7.39	6.6E-03	2.7E-02	Verrucomicrobiota	Candidatus_Udaeobacter
OTU_1732	-3.56	5.47	9.88	1.7E-03	9.9E-03	Verrucomicrobiota	Chthoniobacter
OTU_1080	-2.87	6.31	10.31	1.3E-03	8.4E-03	Verrucomicrobiota	Chthoniobacter
OTU_1792	3.58	5.02	6.61	1.0E-02	3.7E-02	Verrucomicrobiota	Chthoniobacter
OTU_855	-3.32	6.56	9.45	2.1E-03	1.2E-02	Verrucomicrobiota	Chthoniobacter
OTU_1750	-5.06	5.07	11.66	6.4E-04	5.1E-03	Verrucomicrobiota	Methylacidiphilaceae
OTU_2330	-4.34	4.9	7.81	5.2E-03	2.2E-02	Verrucomicrobiota	Methylacidiphilaceae
OTU_535	-2.8	9.38	10.11	1.5E-03	9.1E-03	Verrucomicrobiota	Methylacidiphilaceae
OTU_5632	-2.71	8.02	10.48	1.2E-03	7.9E-03	Verrucomicrobiota	Methylacidiphilaceae
OTU_758	-2.77	7.67	7.45	6.3E-03	2.6E-02	Verrucomicrobiota	Methylacidiphilaceae
OTU_780	-2.75	6.92	10.2	1.4E-03	8.7E-03	Verrucomicrobiota	Methylacidiphilaceae
OTU_956	-4.03	6.97	7.56	6.0E-03	2.5E-02	Verrucomicrobiota	Methylacidiphilaceae
OTU_170	-3.79	9.16	9.89	1.7E-03	9.9E-03	Verrucomicrobiota	Opitutaceae
OTU_1707	-3.79	5.6	8.77	3.1E-03	1.5E-02	Verrucomicrobiota	Opitutaceae
OTU_353	-3.27	8.9	9.46	2.1E-03	1.2E-02	Verrucomicrobiota	Opitutaceae
OTU_442	-4.95	8.85	15.09	1.0E-04	1.5E-03	Verrucomicrobiota	Opitutaceae
OTU_1355	-6.85	8.63	44.55	2.5E-11	2.7E-08	Verrucomicrobiota	Pedosphaeraceae
OTU_150	-3.72	8.44	7.49	6.2E-03	2.6E-02	Verrucomicrobiota	Pedosphaeraceae
OTU_2363	-4.39	7.23	23.02	1.6E-06	8.7E-05	Verrucomicrobiota	Pedosphaeraceae
OTU_3139	-6.56	7.21	27.84	1.3E-07	1.5E-05	Verrucomicrobiota	Pedosphaeraceae
OTU_320	-4.96	8.61	32.68	1.1E-08	2.9E-06	Verrucomicrobiota	Pedosphaeraceae
OTU_3205	-5.56	6.77	18.45	1.7E-05	4.2E-04	Verrucomicrobiota	Pedosphaeraceae

OTU_3214	-6.02	7.06	18.76	1.5E-05	3.8E-04	Verrucomicrobiota	Pedosphaeraceae
OTU_3707	-6.44	7.71	21	4.6E-06	1.6E-04	Verrucomicrobiota	Pedosphaeraceae
OTU_3994	-7.4	6.2	20.34	6.5E-06	2.1E-04	Verrucomicrobiota	Pedosphaeraceae
OTU_745	-3.67	7.42	8.89	2.9E-03	1.5E-02	Verrucomicrobiota	Pedosphaeraceae
OTU_86	-6.35	11.05	42.91	5.7E-11	4.6E-08	Verrucomicrobiota	Pedosphaeraceae
OTU_946	-4.43	6.9	21.84	3.0E-06	1.2E-04	Verrucomicrobiota	Pedosphaeraceae
OTU_118	-7.52	10.68	46.94	7.3E-12	1.2E-08	Verrucomicrobiota	Pedosphaeraceae
OTU_169	-6.87	9.27	36.04	1.9E-09	7.8E-07	Verrucomicrobiota	Pedosphaeraceae
OTU_2491	-6.69	6.69	22.52	2.1E-06	9.5E-05	Verrucomicrobiota	Pedosphaeraceae
OTU_293	-5.18	8.59	24.02	9.5E-07	5.9E-05	Verrucomicrobiota	Pedosphaeraceae
OTU_3185	-5.4	10.19	28.46	9.5E-08	1.3E-05	Verrucomicrobiota	Pedosphaeraceae
OTU_3801	-5.16	6.47	12.63	3.8E-04	3.7E-03	Verrucomicrobiota	Pedosphaeraceae
OTU_70	-6.14	11.62	54.15	2.0E-13	6.0E-10	Verrucomicrobiota	Pedosphaeraceae
OTU_1363	-7.04	5.87	12.51	4.0E-04	3.8E-03	Verrucomicrobiota	Pedosphaeraceae
OTU_1006	-4.79	5.81	8.05	4.5E-03	2.0E-02	Verrucomicrobiota	Pedosphaeraceae
OTU_132	-3.34	8.94	12.86	3.4E-04	3.4E-03	Verrucomicrobiota	Pedosphaeraceae
OTU_1687	-5.13	8.24	39.66	3.0E-10	1.6E-07	Verrucomicrobiota	Pedosphaeraceae
OTU_2239	-4.47	5	9.49	2.1E-03	1.2E-02	Verrucomicrobiota	Pedosphaeraceae
OTU_258	-5.08	9.04	28.19	1.1E-07	1.3E-05	Verrucomicrobiota	Pedosphaeraceae
OTU_1130	5.29	5.72	6.35	1.2E-02	4.1E-02	Verrucomicrobiota	Luteolibacter
OTU_3618	5.6	6.02	13	3.1E-04	3.2E-03	Verrucomicrobiota	Luteolibacter
OTU_828	8.7	9.58	26.6	2.5E-07	2.4E-05	Verrucomicrobiota	Luteolibacter
OTU_1324	5.57	7.81	15.88	6.8E-05	1.1E-03	Verrucomicrobiota	Luteolibacter
OTU_1334	6.3	7.88	19.21	1.2E-05	3.3E-04	Verrucomicrobiota	Luteolibacter
OTU_306	7.81	9.64	19.05	1.3E-05	3.5E-04	Verrucomicrobiota	Luteolibacter
OTU_318	4.54	8.76	15.47	8.4E-05	1.3E-03	Verrucomicrobiota	Luteolibacter
OTU_4462	5.41	5.26	7.5	6.2E-03	2.5E-02	Verrucomicrobiota	Luteolibacter
OTU_45	9.23	12.49	19.16	1.2E-05	3.4E-04	Verrucomicrobiota	Luteolibacter
OTU_571	7.82	9.29	21.96	2.8E-06	1.2E-04	Verrucomicrobiota	Luteolibacter
OTU_111	-2.91	9.32	9.75	1.8E-03	1.0E-02	WPS-2	WPS-2
OTU_434	-3.59	6.62	6.2	1.3E-02	4.4E-02	WPS-2	WPS-2
OTU_555	-3.03	7.05	6.8	9.1E-03	3.4E-02	WPS-2	WPS-2

OTU_5727	-2.77	7.56	10.34	1.3E-03	8.3E-03	WPS-2	WPS-2
OTU_85	-2.32	10.51	15.16	9.9E-05	1.5E-03	WPS-2	WPS-2
OTU_977	-2.19	5.97	6.07	1.4E-02	4.6E-02	WPS-2	WPS-2
OTU_354	5.49	8.4	8.11	4.4E-03	2.0E-02	WPS-2	WPS-2
OTU_680	4.87	6.99	9.66	1.9E-03	1.1E-02	WPS-2	WPS-2

Supplementary Table S4 - Average, median and standart deviation of the nitrogen-cycle genes, and results of their number comparison in different groups of microbial communities performed by Kruskal-Wallis rank sum test and Dunn test Bonferroni; Number of replicates for each group: Unaffected - 5, Impacted - 4, Desolated - 5, Control Dead - 3

Gene	Impact	Median	IQR	Ave-rage	SD	Kruskal-Wallis rank sum test	Dunn test; Unaffected and Desolated groups
ureC	Unaffected	2.8×10^{6}	2.2×10^{6}	3.3×10^{6}	2.4×10^{6}	H = 10.3 df = 3 p = 0.02	D = 3.0 p = 0.0079
	Impacted	5.7×10^{6}	5.8×10^{6}	7.0×10^{6}	5.3×10^{6}		
	Desolated	2.2×10^{7}	6.9×10^{6}	2.2×10^{7}	1.0×10^{7}		
	Control Dead	2.9×10^{6}	4.2×10^{6}	4.9×10^{6}	4.6×10^{6}		
amoA	Unaffected	1.4×10^{3}	5.8×10^{3}	3.7×10^{3}	4.0×10^{3}	H = 9.8 df = 3 p = 0.02	D = 2.9 p = 0.0097
	Impacted	4.1×10^{3}	3.6×10^{3}	6.6×10^{3}	5.4×10^{3}		
	Desolated	2.8×10^{4}	1.5×10^{4}	2.7×10^{4}	1.5×10^{4}		
	Control Dead	6.2×10^{3}	1.9×10^{3}	5.4×10^{3}	2.0×10^{3}		
Nitro-spira clade A, *amoA*	Unaffected	1.2×10^{4}	2.4×10^{4}	1.9×10^{4}	1.3×10^{4}	H= 9.8 df = 3 p = 0.02	D = 2.9 p = 0.0097
	Impacted	2.2×10^{4}	2.2×10^{4}	2.4×10^{4}	1.7×10^{4}		
	Desolated	7.5×10^{4}	9.1×10^{3}	7.2×10^{4}	1.7×10^{4}		
	Control Dead	2.2×10^{4}	6.8×10^{3}	2.6×10^{4}	7.8×10^{3}		
Nitro-spira clade B, *amoA*	Unaffected	6.9×10^{4}	4.9×10^{4}	9.5×10^{4}	8.5×10^{4}	H = 10.4 df = 3 p = 0.02	D = 2.9 p = 0.01
	Impacted	1.7×10^{5}	1.2×10^{5}	1.6×10^{5}	9.3×10^{4}		
	Desolated	4.6×10^{5}	1.5×10^{5}	4.2×10^{5}	1.0×10^{5}		
	Control Dead	6.7×10^{4}	4.3×10^{4}	8.8×10^{4}	4.7×10^{4}		
nirS	Unaffected	4.3×10^{7}	7.4×10^{7}	5.4×10^{7}	4.4×10^{7}	H = 7.8 df = 3 p = 0.05	D = 2.6 p = 0.02
	Impacted	9.5×10^{7}	6.6×10^{7}	9.5×10^{7}	5.8×10^{7}		
	Desolated	2.3×10^{8}	3.0×10^{7}	2.2×10^{8}	1.0×10^{8}		
	Control Dead	5.1×10^{7}	5.1×10^{7}	7.5×10^{7}	5.6×10^{7}		

Supplementary Material

nxrB	Unaffected	1.7×10^4	1.4×10^4	3.7×10^4	5.6×10^4		
	Impacted	4.6×10^4	4.7×10^4	5.2×10^4	3.8×10^4		
	Desolated	1.3×10^5	3.5×10^4	1.4×10^5	7.6×10^4		
	Control Dead	1.9×10^4	1.0×10^4	2.4×10^4	1.1×10^4	$H = 7.7$<br>$df = 3$<br>$p = 0.05$	-
nosZ	Unaffected	5.2×10^6	3.9×10^6	5.0×10^6	3.4×10^6		
	Impacted	9.5×10^6	6.0×10^6	1.0×10^7	7.7×10^6		
	Desolated	3.1×10^7	4.9×10^6	4.0×10^7	2.4×10^7		
	Control Dead	7.0×10^6	3.5×10^6	6.7×10^6	3.5×10^6	$H = 10.4$<br>$df = 3$<br>$p = 0.02$	$D = 3.1$<br>$p = 0.006$
16S rRNA	Unaffected	1.6×10^7	2.7×10^7	1.9×10^7	1.5×10^7		
	Impacted	6.7×10^7	8.1×10^7	6.5×10^7	5.2×10^7		
	Desolated	2.0×10^8	1.7×10^8	2.5×10^8	1.2×10^8		
	Control Dead	1.4×10^7	5.2×10^6	1.7×10^7	5.7×10^6	$H = 10.9$<br>$df = 3$<br>$p = 0.01$	$D = 2.9$<br>$p = 0.0097$
